# Interferon signaling is required for early neutrophil recruitment after zebrafish heart injury

**DOI:** 10.1242/bio.062374

**Published:** 2026-06-15

**Authors:** Alexis V. Schmid, James A. Gagnon

**Affiliations:** ^1^ School of Biological Sciences, University of Utah, Salt Lake City, UT, 84112, USA; ^2^ Henry Eyring Center for Cell & Genome Science, University of Utah, Salt Lake City, 84112, USA

**Keywords:** Heart regeneration, Interferon, Zebrafish, Cryoinjury, Neutrophils

## Abstract

Heart injury triggers a robust cellular response in zebrafish, characterized by neovascularization, immune cell activation, and the infiltration of proliferative cardiomyocytes that collectively lead to scarless regeneration. Upon injury, damage-associated molecular patterns are released by dying cells and injured extracellular matrix. These molecules bind to pattern recognition receptors on various cell types, promoting inflammation and immune cell recruitment by upregulating chemokines and pro-inflammatory cytokines. We previously identified the activation of injury-induced interferon signaling as a distinguishing feature between the regenerative zebrafish and non-regenerative medaka. Here, we establish interferon-Φ1 (IFNφ1) as the primary driver of interferon signaling after zebrafish heart injury. IFNφ1 expression is induced hours after injury and directs interferon-stimulated gene expression, which peaks at 3 days post-injury. This response is lost in *ifnphi1* mutants, which disrupt IFNφ1 expression. *ifnphi1* mutants have reduced neutrophil recruitment to the injured myocardium, while macrophages and neovascularization are unaffected. By studying later stages of regeneration, we find that *ifnphi1* mutant hearts have a modest defect in fibrotic tissue resolution. Collectively, these findings uncover a critical early signaling cascade during the inflammatory response to heart injury and provide new insights into the mechanisms that choreograph zebrafish heart regeneration.

## Teaser

**Summary:** Interferon-Φ1 signaling is required for early neutrophil recruitment after zebrafish heart injury, revealing a key immune trigger that shapes inflammation and may influence efficient cardiac regeneration.

## INTRODUCTION

Myocardial infarction occurs when blood flow to a portion of the heart is blocked, leading to irreparable cell death and the formation of a large, inelastic scar that hinders heart function (Salari et al., 2023). Nearly all mammals lose their regenerative capacity after the neonatal stage, whereas most fish and amphibians retain it throughout development and into adulthood. These regenerators replace collagenous fibrotic tissue with injury-responsive cardiomyocytes to restore heart function to the injured myocardium (González-Rosa et al., 2011, 2017; Poss et al., 2002). Zebrafish have emerged as a powerful model to study cardiac regeneration (Gemberling et al., 2013). Following cardiac injury, zebrafish employ a sequence of pro-inflammatory and anti-inflammatory immune signals to coordinate collagen deposition, revascularization, and eventual resolution of fibrotic tissue (Marín-Juez et al., 2016; Sánchez-Iranzo et al., 2018). In contrast, the non-regenerating medaka fish exhibit a prolonged pro-inflammatory immune response after injury, leading to scar persistence (Lai et al., 2017). Studies of regenerators may yield new therapeutic insights into the specific immune and cellular mechanisms required to resolve fibrotic tissue and regenerate functional cardiac tissue.

In zebrafish, heart regeneration is initiated by a choreographed immune response. The initial injury triggers the release of damage-associated molecular patterns (DAMPs) from necrotic tissue and injured extracellular matrix. These DAMPs induce inflammation by binding to pattern recognition receptors (PRRs) on multiple cell types to recruit waves of immune cells to the wound via the release of pro-inflammatory cytokines and chemoattractants (Iribarne, 2021; Julier et al., 2017). Neutrophils are the first pro-inflammatory cells recruited to the injury zone, rapidly localizing to the wound as early as 6 h post-injury (hpi) (Julier et al., 2017). Neutrophils are vital for wound detection by helping to recruit monocytes, which differentiate into macrophages at the wound site (Feng et al., 2025; Li et al., 2012; Wilgus et al., 2013). In addition to promoting inflammation early after injury, neutrophils also secrete vascular endothelial growth factor (VEGF-A), which promotes early neovascularization of the injured myocardium (El-Sammak et al., 2022; Gong and Koh, 2010; Lörchner et al., 2023). Neutrophils also activate epicardial cells to induce epithelial-to-mesenchymal transition, enabling epicardial infiltration and significant contributions to wound healing (Peterson et al., 2024).

Macrophages are the second immune cells recruited to the injury site, providing both a pro-inflammatory and an anti-inflammatory response (Bevan et al., 2020; Sánchez-Iranzo et al., 2018). The numerous roles of macrophages are still being explored; however, we know they shape the inflammatory response by phagocytosing pro-inflammatory apoptotic neutrophils, clearing cellular debris, shaping new extracellular matrix and neovasculature, and depositing collagen alongside fibroblasts for formation of fibrotic tissue (Bevan et al., 2020; Constanty et al., 2025; Goumenaki et al., 2024; Julier et al., 2017). While immune cells are required for complete regeneration, the specific immune responses that distinguish regenerating and non-regenerating species are still being investigated (Dolejsi et al., 2022; Lai et al., 2017; Peterson et al., 2024).

We previously compared the response to heart injury in two distantly related teleost species, medaka and zebrafish, with a common ancestor ∼140 million years ago. Despite these fish having similar heart morphology and gene orthology, medaka cannot recover injured myocardium. The innate immune landscape in medaka is different from that of zebrafish, with higher neutrophil recruitment, less macrophage recruitment, and an overall more pro-inflammatory response (Lai et al., 2017). Interestingly, stimulating macrophage recruitment with the TLR agonist, Poly I:C, stimulates heart regeneration in medaka, indicating a failure in the initial damage-sensing phase (Lai et al., 2017). However, the specific signaling pathways underlying this process in zebrafish remain unclear. Using single-cell RNA sequencing, we identified a key difference in the inflammatory response after injury. We found that medaka lacked an injury-induced interferon signaling response to injury. Conversely, zebrafish display robust upregulation of interferon-stimulated genes (ISGs) across most cardiac cell types at three days post-injury (dpi) during the inflammatory stage (Carey et al., 2024).

Interferons are pro-inflammatory cytokines that are released by immune cells responding to infection or injury (Cheng et al., 2008). There are three types of interferons in zebrafish: type I (IFNΦ1-4), type II (IFNγ1-2), and type IV (IFNυ) (Aggad et al., 2009; Chen et al., 2022; Cheng et al., 2008). IFNγ studies in mice and in cell culture have highlighted the importance of type II interferon signaling in macrophage activation and successful skeletal muscle regeneration. However, prolonged IFNγ exposure in these models led to excessive fibrosis, reduced muscle contractility, and inhibited regeneration (Adhikari et al., 2020; Chen et al., 2021; Cheng et al., 2008). Our single-cell RNA sequencing analysis of injured zebrafish and medaka hearts revealed specific upregulation of *ifnphi1* in injured zebrafish hearts (Carey et al., 2024). Still, the role of type I interferon signaling in heart regeneration remains unexamined. Interferons bind to various cell types in the heart, which leads to the expression of ISGs. ISGs have defined roles in pathogen clearance by recruiting innate immune cells to the infection (Balla et al., 2020; Schneider et al., 2014); however, whether ISGs play a role in coordinating a pro-regenerative immune response during heart regeneration is unclear.

Here, we establish IFNφ1 as the principal driver of interferon signaling following heart injury in zebrafish. *ifnphi1* gene expression is rapidly induced at the injury site, pointing to a role in early damage sensing and immune cell recruitment. We find that interferon signaling is required to recruit neutrophils to the injury site, an important early step for creating a pro-regenerative environment. Despite this key role in early inflammation, *ifnphi1^−/−^* showed no defects in later regenerative processes, including macrophage recruitment and neovascularization. However, injured *ifnphi1^−/−^* hearts still had a modest defect in resolution of fibrotic tissue. Overall, *ifnphi1*-driven interferon signaling orchestrates a discrete, essential role in the initiation of inflammation without being requisite for the subsequent complete regeneration of the zebrafish heart.

## RESULTS

### Interferon signaling is an immediate and injury-specific pathway activated during the inflammatory response

**Fig. 1.**
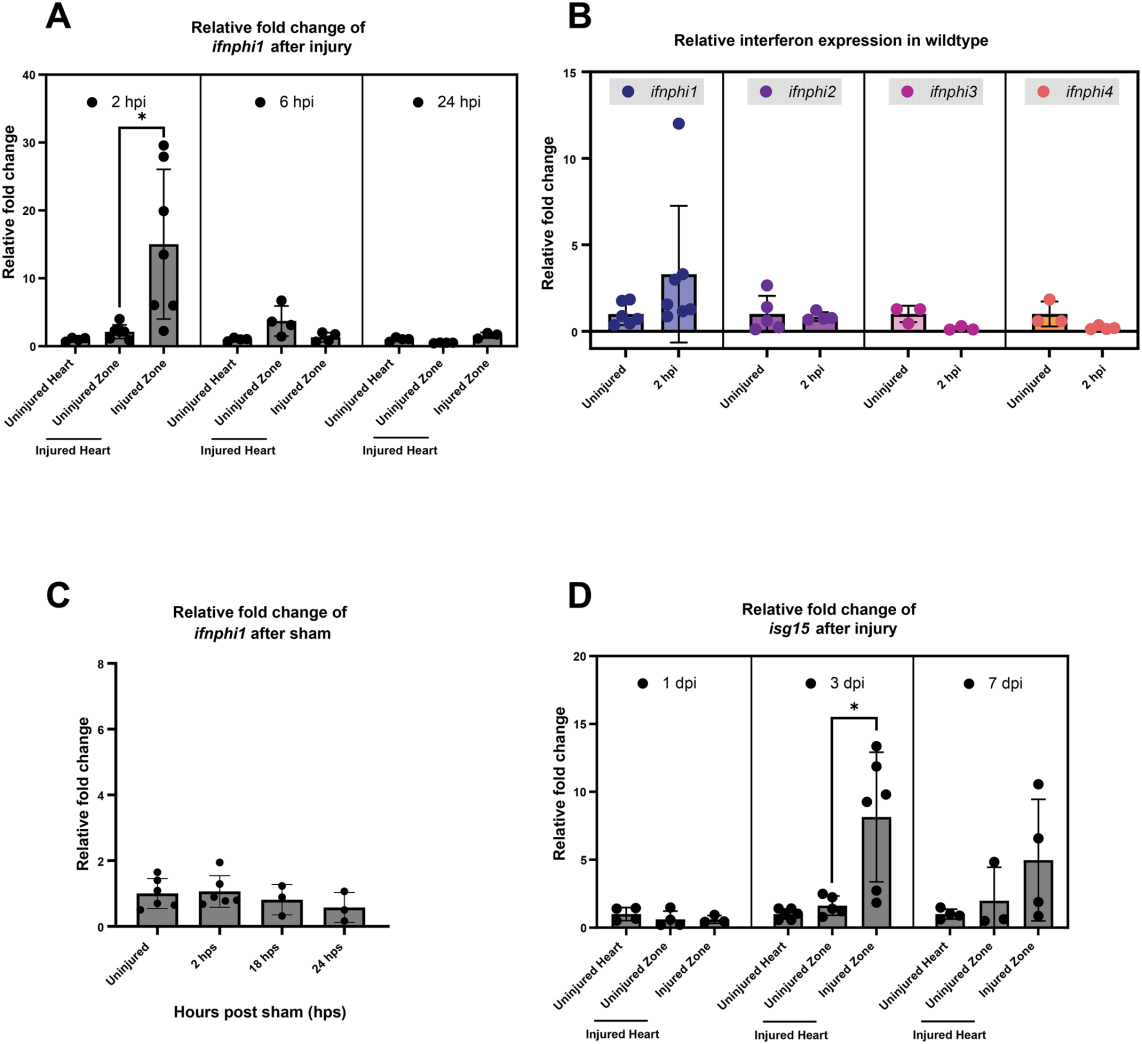
**Interferon signaling is induced by zebrafish heart injury.** (A) A time course of *ifnphi1* gene expression after cryoinjury 2, 6, and 24 hpi normalized to *elf1a* expression. Relative fold change calculated by normalizing to uninjured control ventricles. Each dot on the plot represents a pool of three uninjured or three injured zones of injured ventricles. Welch's *t*-test was used to determine statistical significance between the relative fold change of *ifnphi1* in the injured zone relative to the uninjured zone, with *P=*0.010 at 2 hpi. (B) *ifnphi1-4* gene expression in wild-type ventricles at 2 hpi relative to uninjured ventricles. (C) A time course of *ifnphi1* gene expression after sham injury 2, 18, and 24 h post sham (hps) relative to uninjured ventricles. (D) A time course of *isg15* gene expression after cryoinjury 1, 3, and 7 dpi relative to uninjured ventricles. Welch's *t*-test was used to determine statistical significance between the relative fold change of *isg15* in the injured zone relative to the uninjured zone, with *P=0.019 at 3 dpi. For A and D, each dot on the plot represents a pool of three uninjured or three injured zones of injured ventricles. For B and C, each dot shown represents a single ventricle after dissection away from the atrium and bulbus arteriosus. Data are shown as the mean±s.d.

We previously identified ISGs as significantly upregulated 3 dpi in zebrafish endocardial, epicardial, and leukocyte cell populations within the ventricle, and coincide with an increase in *ifnphi1*-positive endothelial cells (Carey et al., 2024). In contrast, non-regenerative medaka lack an injury-induced interferon response, suggesting that interferon signaling may play an important role in creating a pro-regenerative environment in zebrafish early after heart injury. To better characterize this signaling pathway in zebrafish, we examined the transcript levels of *ifnphi1* mRNA in isolated injured and uninjured zones of zebrafish ventricles over a time course following experimental cryoinjury (Fig. 1A). Relative to the uninjured control hearts, *ifnphi1* expression significantly peaked 2 h after cryoinjury and returned to baseline expression levels by 24 hpi (*P=*0.010, Fig. 1A). *ifnphi*2-4 transcript levels were unchanged in injured hearts relative to uninjured hearts driving our focus on investigating *ifnphi1* (Fig. 1B). Given the role of interferon signaling in pathogen clearance, and the common presence of infectious agents in fish facility water (Balla et al., 2020), *ifnphi1* upregulation was also tested after sham injury. This injury exposes the fish's chest cavity to the fish facility water without damaging the ventricle. Here, no upregulation of *ifnphi1* was observed, providing evidence that interferon expression is specifically responding to heart cryoinjury rather than to chest cavity exposure (Fig. 1C).

We previously reported that *isg15* and other genes induced by interferon signaling are expressed 3 dpi in endothelial, epicardial, and leukocyte cell types (Carey et al., 2024). To further characterize the production of ISGs downstream of interferon signaling, we used RT-qPCR to measure the relative fold change of *interferon-stimulated gene-15* (*isg15*) mRNA in isolated injured and uninjured zones of zebrafish ventricles over a time course following cryoinjury. Relative to uninjured hearts, *isg15* expression peaks at 3 dpi, well after the initial expression of interferon mRNA (*P=*0.019, Fig. 1D). Together, these data describe the tempo of interferon signaling after zebrafish heart injury: induced immediately after injury and received by endocardial, epicardial, and leukocyte cells to promote the expression of interferon-stimulated genes within the first 3 days after injury.

### Generating an *ifnphi1* mutant zebrafish

**Fig. 2.**
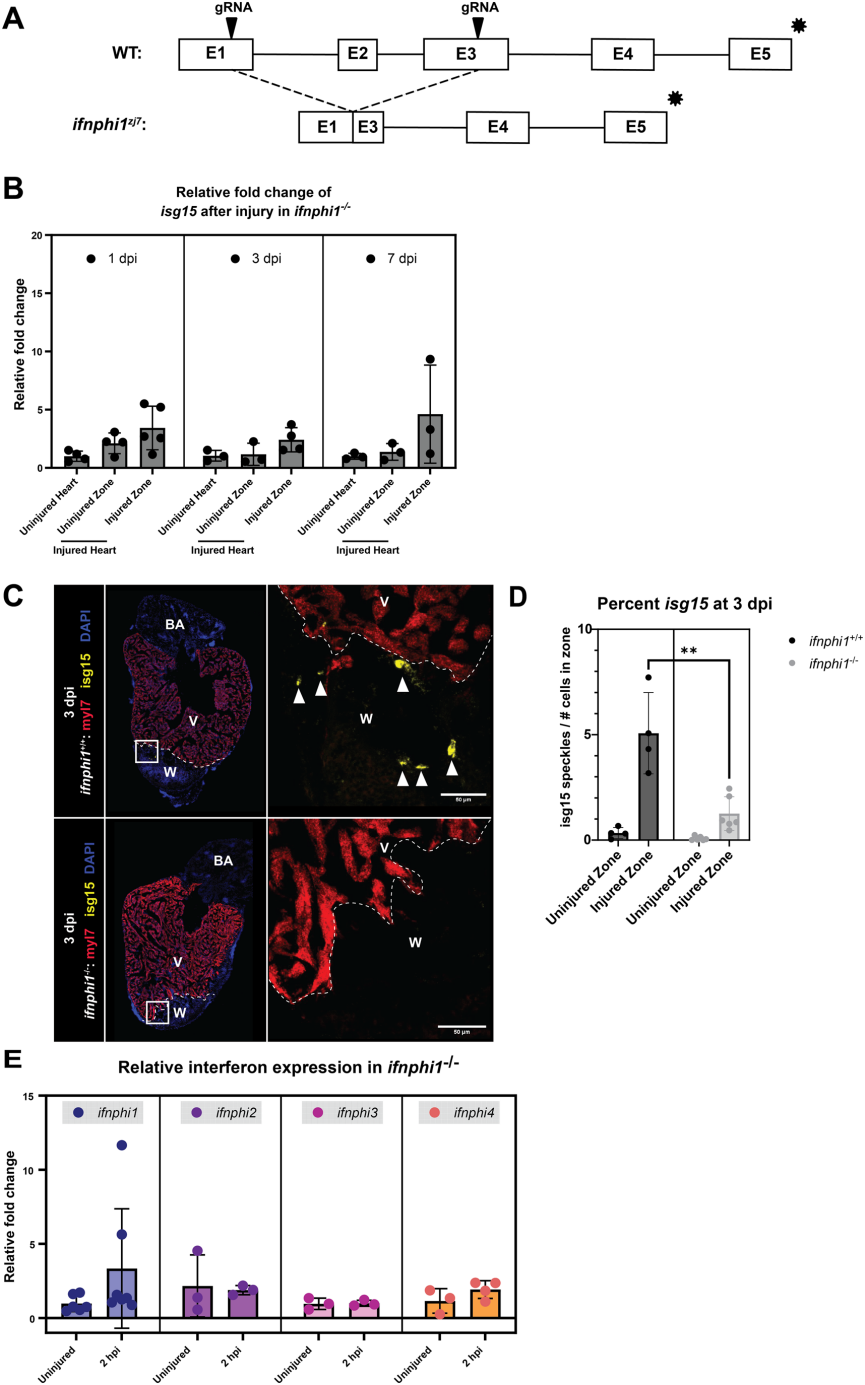
**A zebrafish mutant in *ifnphi1* disrupts interferon signaling after heart injury.** (A) A design for mutating the *ifnphi1* gene, with flanking guide RNAs shown (above) and the *ifnphi*^zj7^ allele with a large deletion that removes part of exon 1, all of exon 2, and part of exon 3 (see Table S1 for sequences). (B) A time course of *isg15* gene expression after cryoinjury in *ifnphi1^−/−^* ventricles 1, 3, and 7 dpi relative to uninjured ventricles. Each dot on the plot represents a pool of three uninjured or three injured zones of injured ventricles. (C) Representative images of RNA fluorescence *in situ* hybridization (RNA-FISH) of *isg15* (interferon stimulated gene-15) and *myl7* (labels cardiomyocytes) in wild-type and *ifnphi1^−/−^* ventricle cryosections at 3 dpi. Sections were counterstained with DAPI (blue). BA, bulbus arteriosus; V, uninjured ventricle; W, wound. Injury border is shown as a dashed line. (D) Percent *isg15* calculated via the number of *isg15* speckles quantified in CellProfiler divided by the number of nucleated cells in the injured and uninjured zones. Welch's *t*-test was used to determine statistical significance between the *isg15* speckle percent in the injured zones (*P*=0.0023). The dots on the graph represent the uninjured or injured zones of individual ventricles. Data are shown as the mean±s.d.; *n*=4 wild-type and *n*=6 *ifnphi1^−/−^* ventricles. Scale bars: 50 μm. (E) *ifnphi1-4* gene expression in *ifnphi1^−/−^* ventricles at 2 hpi relative to uninjured ventricles.

To study the role of interferon signaling in heart regeneration, we generated an *ifnphi1^−/−^* zebrafish. Wild-type embryos were injected with four gRNAs, two each targeting exon 1 and exon 3 of the *ifnphi1* gene. Through crossing, we established a stable line with a large in-frame deletion between these target sites, which should not induce NMD-mediated degradation of the mRNA but is predicted to disrupt protein function by deleting >30% of amino acids from the mature protein (Fig. 2A; Table S1). We assessed the functional disruption of interferon signaling in the mutant using RT-qPCR to evaluate *isg15* expression in injured mutant and wild-type ventricles. *ifnphi1^−/−^* fish had no induction of *isg15* mRNA after injury, lacking the *isg15* upregulation at 3 dpi observed in the injured zones of wild-type ventricles (Fig. 2B). As an orthogonal method to confirm that our mutant disrupted interferon signaling in the heart after injury, we visualized the production of *isg15* transcripts using RNAscope at 3 dpi in *ifnphi1^−/−^* and *ifnphi1^+/+^* hearts. This experiment revealed that *ifnphi1^+/+^* zebrafish had enrichment of *isg15* mRNA in the injured zone of their ventricles, while *ifnphi1^−/−^* fish lacked *isg15* enrichment in the injured myocardium (Fig. 2C,D; Fig. S1). Based on the loss of *isg15* expression after injury, we infer that our *ifnphi1* mutant allele is a loss-of-function allele.

To ensure that the other type I interferons were not compensating for the lack of *ifnphi1* in mutants, RT-qPCR was used to test for the upregulation of *ifnphi2-4* in both *ifnphi1^+/+^* and *ifnphi1^−/−^* fish, relative to uninjured *ifnphi1^+/+^* fish (Fig. 2E). Here, *ifnphi2-4* is not upregulated in *ifnphi1^+/+^* or *ifnphi1^−/−^* conditions after injury. Note that the qPCR primers for *ifnphi1* amplify a region spanning exons 4 and 5, outside the deleted area in our mutant allele; hence, a peak in *ifnphi1^−/−^* fish at 2 hpi is expected. Collectively, these experiments provide strong evidence that *ifnphi1* is the primary driver of interferon signaling after cardiac injury in zebrafish, and that *ifnphi1^−/−^* fish have disrupted interferon signaling.

### *ifnphi1* is required for early neutrophil recruitment to the injured myocardium

**Fig. 3.**
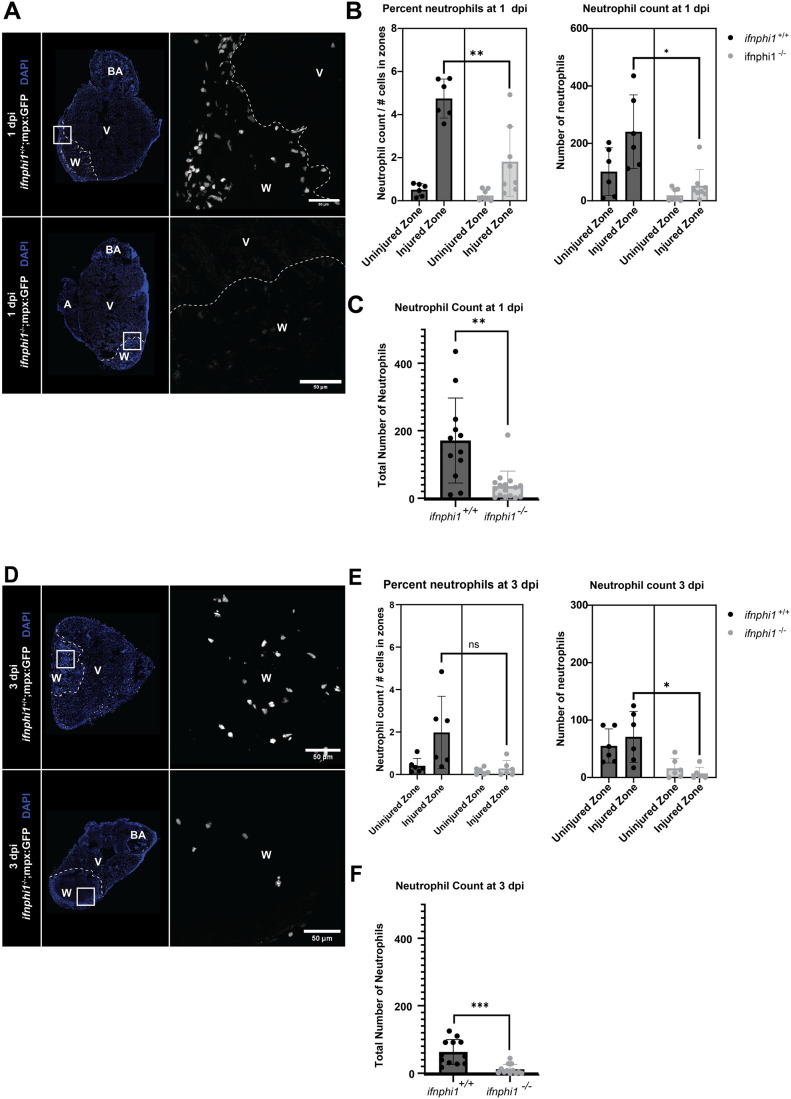
**Interferon signaling promotes neutrophil recruitment to injured myocardium.** (A) Representative images of wild-type Tg(mpx:GFP) and *ifnphi1^−/−^* Tg(mpx:GFP) heart sections at 1 dpi. Sections were counterstained with DAPI (blue). Neutrophils are shown in white. Injury border is shown as a dashed line. (B) Quantification of the percent of neutrophils and absolute number of neutrophils in the injured and uninjured zones of wild-type and *ifnphi1^−/−^*hearts at 1 dpi. The total number of neutrophils was counted in the uninjured and injured zones in wild-type and *ifnphi1^−/−^* hearts (*P*=0.0137). The percent of neutrophils was calculated by dividing the number of neutrophils by the number of nucleated cells in the uninjured and injured zones in wild-type and *ifnphi1^−/−^* hearts (*P*=0.0012). (C) The sum of neutrophils in uninjured and injured zones in wild-type and *ifnphi1^−/−^* hearts at 1 DPI (*P=*0.0035). (D) Representative images of wild-type Tg(mpx:GFP) and *ifnphi1^−/−^* Tg(mpx:GFP) heart sections at 3 dpi. Sections were counterstained with DAPI (blue). Injury border is shown as a dashed line. (E) Quantification of the percent of neutrophils (*P*=0.0590) and absolute number of neutrophils (*P*=0.0157) in the injured and uninjured zones of wild-type and *ifnphi1^−/^^−^* hearts at 1 dpi was calculated as above. (F) The sum of neutrophils in uninjured and injured zones in wild-type and *ifnphi1^−/−^* hearts at 3 dpi (*P=*0.0005). Each dot on the graphs represents the uninjured or injured zones calculated from individual sections of distinct heart ventricles. Data are shown as the mean±s.d.; *n*=6 wild type and *n*=6 *ifnphi1^−/−^* for 1 dpi, and *n*=6 wild type and *n*=6 *ifnphi1^−/^^−^* for 3 dpi. BA, bulbus arteriosus; V, uninjured ventricle; W, wound. Statistical tests represent Welch's *t*-test. Scale bars: 50 μm.

Given the strong connections between interferon signaling and innate immune cell recruitment, we next examined the role of *ifnphi1* in neutrophil and macrophage recruitment to the injury site. To study the role of interferon signaling in neutrophil recruitment, we labeled neutrophils with the mpx:GFP transgene in wild-type and ifnphi1^−/−^ hearts. Neutrophils are recruited to the heart only after injury via the vasculature, promoting inflammation at the injured site (Ma et al., 2013; Peterson et al., 2024). Neutrophils are the first innate immune responders, arriving in the injured zone as early as 6 hpi and peaking at 1 dpi (Goumenaki et al., 2024; Julier et al., 2017). We dissected and imaged injured hearts at 1 dpi. Because the impact of cryoinjury can vary between hearts, we calculated the total number of neutrophils and the percentage of neutrophils in the injured and uninjured zones by dividing the total number of neutrophils by the total number of nucleated cells in each injury zone (Fig. 3A,B). By both metrics, *ifnphi1^−/−^* fish had significantly fewer neutrophils in the injury zone than *ifnphi1^+/+^* fish at 1 dpi. Furthermore, the cumulative number of neutrophils was counted in the uninjured and injured zones in both *ifnphi1^−/−^* and *ifnphi^+/+^* fish. At 1 dpi, *ifnphi^+/+^* fish had significantly more neutrophils recruited to the heart overall than *ifnphi1^−/−^* fish, suggesting a defect in overall neutrophil recruitment after injury (*P=*0.0035, Fig. 3C).

To observe if this defect persisted, we conducted the same comparison at 3 dpi. While 3 dpi is after the peak neutrophil response in the injury zone, *ifnphi1^+/+^* fish still had a significantly higher number of neutrophils than *ifnphi1^−/−^* fish (*P=*0.0005, Fig. 3D,E). The total number of neutrophils in the uninjured and injured zones in both *ifnphi1^−/−^* and *ifnphi^+/+^* fish also significantly differed (*P=*0.0035, Fig. 3F). These data suggest that interferon signaling is required for the recruitment of neutrophils to the heart, as well as the enrichment of neutrophils in the injured zone during the initial stage of zebrafish heart regeneration.

### Interferon signaling is dispensable for macrophage recruitment and coronary endothelial cell regeneration after heart injury

**Fig. 4.**
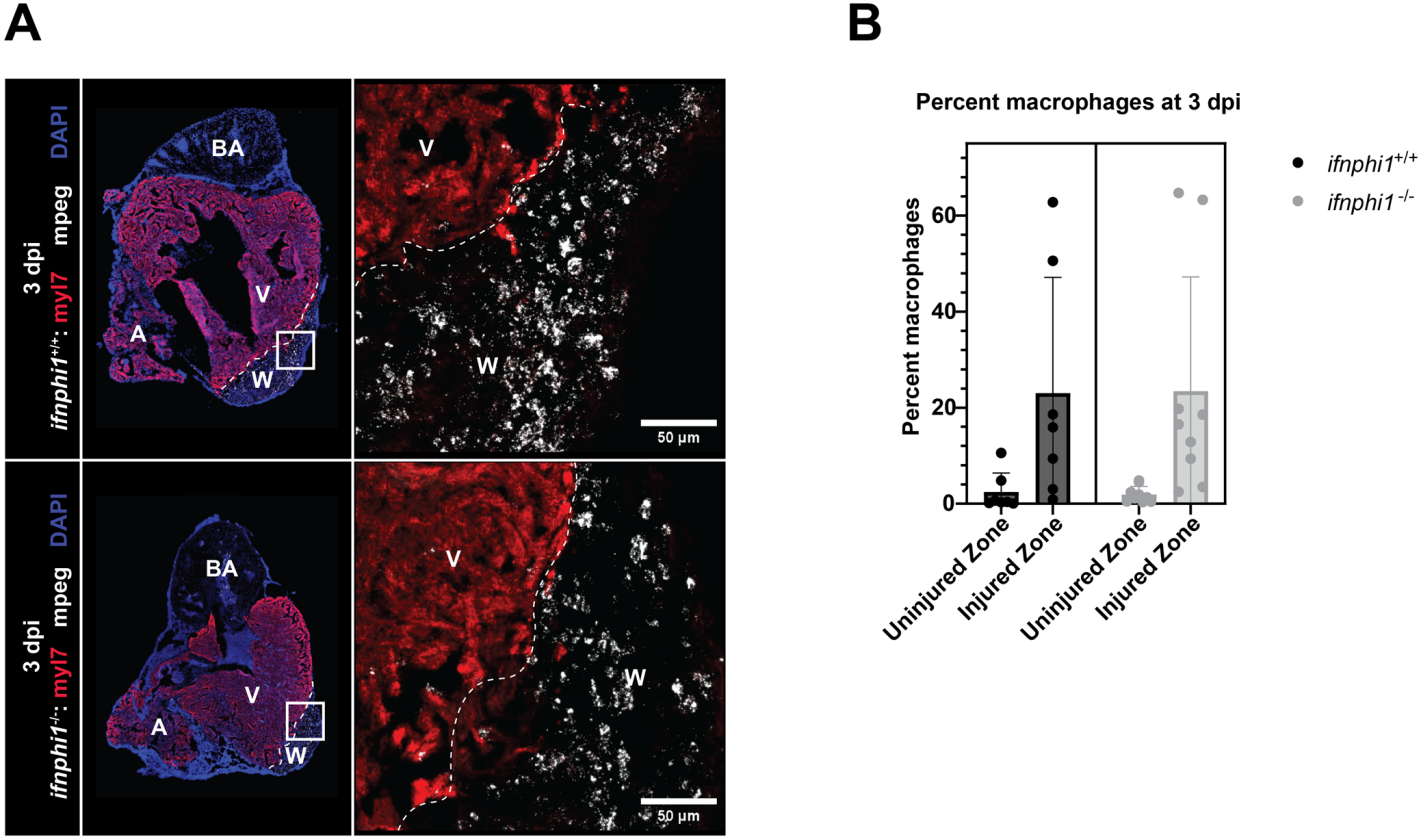
**Interferon signaling is not required for macrophage recruitment to the heart injury zone.** (A) Representative images of RNA-FISH of *mpeg* (labels macrophages) and *myl7* (labels cardiomyocytes), in wild-type and *ifnphi1^−/−^* ventricle cryosections at 3 dpi. Sections were counterstained with DAPI (blue). BA, bulbus arteriosus; A, atrium; V, uninjured ventricle; W, wound. Injury border is shown as a dashed line. (B) Percent macrophages were calculated by dividing the number of *mpeg* speckles by the number of nucleated cells in the injured and uninjured zones. The dots on the graph represent the uninjured or injured zones calculated from individual sections of distinct heart ventricles. Data are shown as the mean±s.d.; *n*=7 wild type and *n*=9 *ifnphi1^−/−^*. Scale bars: 50 μm.

Neutrophils directly interact with macrophages by secreting chemokines and cytokines, thereby influencing the pro-repair, anti-inflammatory identity of infiltrating macrophages (Bouchery and Harris, 2019; Wilgus et al., 2013). In the wound, macrophages phagocytose cellular debris and dying neutrophils, contributing to the transition to an anti-inflammatory immune response (Bouchery and Harris, 2019; Marwick et al., 2018). In addition to shaping the inflammatory response, macrophages closely associate with protruding cardiomyocytes at the border injury zone and help remodel the extracellular matrix to facilitate resolution of fibrotic tissue (Constanty et al., 2025). To estimate the number of macrophages that infiltrated the injury site in wild-type and *ifnphi1*^−/−^ mutant hearts, we measured *mpeg1.1* expression, a classic marker of macrophages, using RNA-FISH (Ellett et al., 2011; Kuil et al., 2020; Carey et al., 2024). We found that both genotypes have similar amounts of mpeg expression (Fig. 4A,B; Fig. S2), suggesting that macrophage abundance was not impacted by the lack of interferon signaling in the mutants. Despite the implications of macrophage recruitment and TLR signaling demonstrated in a previous medaka study, our data suggest that *ifnphi1*-driven interferon signaling does not play a role in macrophage recruitment in zebrafish (Lai et al., 2017) (Fig. 4A,B).

**Fig. 5.**
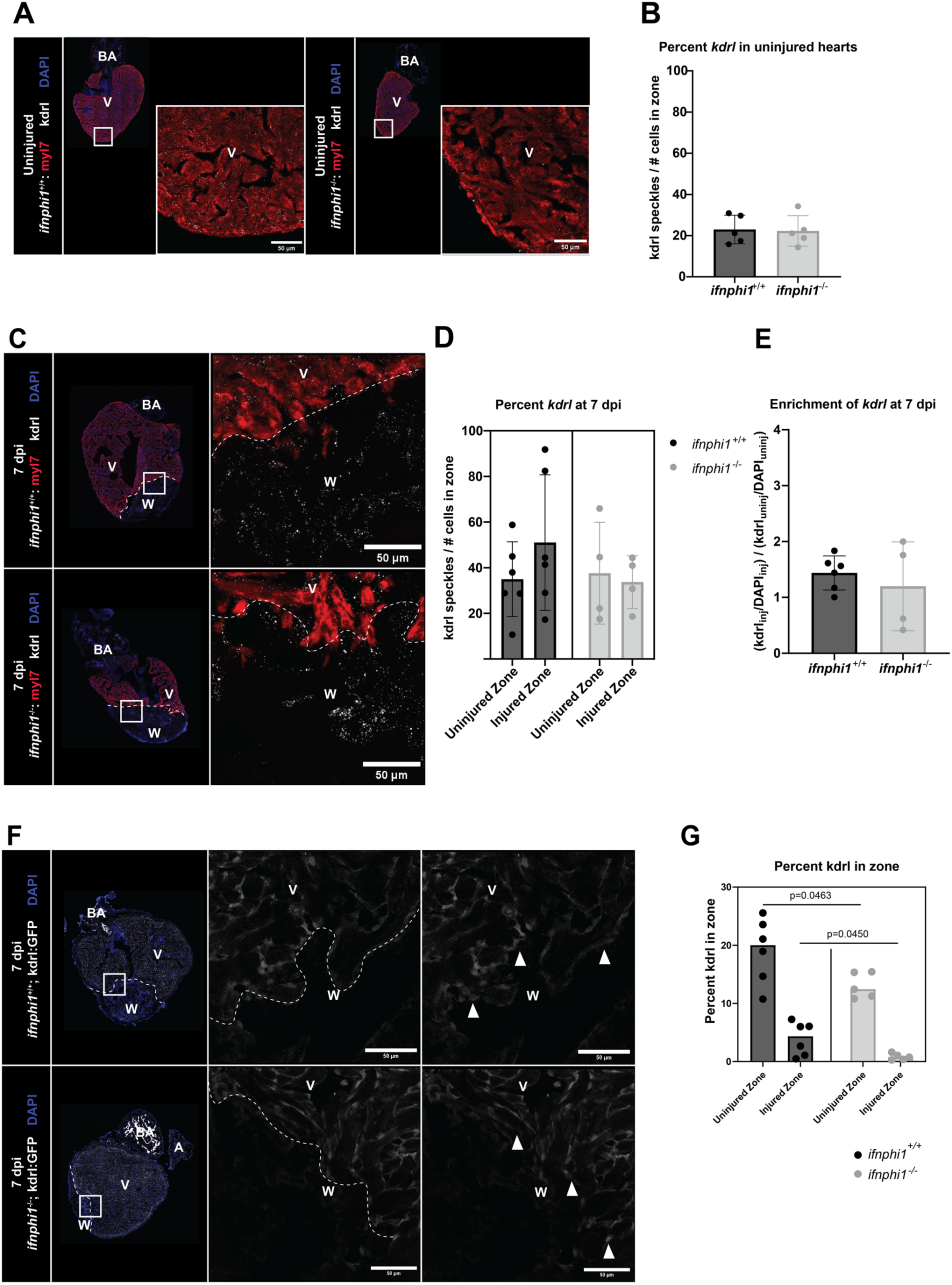
**Interferon signaling is not required for endothelial regeneration after heart injury.** (A,C) Representative images of RNA-FISH of *kdrl* (labels vasculature) and *myl7* (labels cardiomyocytes) in uninjured and 7 dpi wild-type and *ifnphi1^−/−^* ventricle cryosections. Sections were counterstained with DAPI (blue). Injury border is shown as a dashed line. (B,D) Percent *kdrl* was calculated by dividing the number of *kdrl* speckles by the number of nucleated cells in the injured and uninjured zones at each time point. (E) *kdrl* enrichment was calculated by dividing the normalized *kdrl* speckle count in the injured zone by the normalized *kdrl* speckle count in the uninjured zone of the same heart section, for each heart. (F) Representative images of wild-type Tg(kdrl:GFP) and *ifnphi1^−/−^* Tg(kdrl:GFP) heart sections at 7 dpi. Sections were counterstained with DAPI (blue). Cardiac endothelial cells are in white. Injury border is shown as a dashed line. Middle and right panels are kdrl:GFP channel only. Arrowheads indicate modest endothelial regrowth in the injury zone. (G) Percent kdrl pixels in injured or uninjured area. The dots on the graph represent the uninjured or injured zones calculated from individual sections of distinct heart ventricles. BA, bulbus arteriosus; A, atrium; V, uninjured ventricle; W, wound. Data are shown as the mean±s.d.; *n*=5 wild type and *n*=5 *ifnphi1^−/−^* for uninjured samples, *n*=6 wild-type and *n*=4 *ifnphi1^−/−^* for 7 dpi RNAscope samples, and *n*=6 wild-type and *n*=5 *ifnphi1^−/−^* for 7 dpi antibody samples. Scale bars: 50 μm.

Our single-cell data identified an enrichment of *interferon-stimulated gene* expression in the cardiac endothelial cells (Carey et al., 2024), suggesting a potential role for interferon signaling in cardiac endothelial cell regeneration. We measured the cardiac endothelium at uninjured and 7 dpi in *ifnphi1^+/+^* and *ifnphi1^−/−^* hearts using RNA-FISH for *kdrl* expression and the Tg(kdrl:GFP) (Fig. 5) (Lowe et al., 2021; Zhao et al., 2014). The *kdrl* speckles were counted in both injured and uninjured zones of the myocardium and normalized to the number of nucleated cells in each zone (Fig. 5B,D). The enrichment of *kdrl* expression in the injured versus uninjured zones was calculated for each heart (Fig. 5E). Additionally, the number of *kdrl* speckles in the uninjured and injured zones for each heart was totaled (Fig. S3). We found that uninjured *ifnphi1^+/+^* and *ifnphi1^−/−^* hearts had very similar *kdrl* expression (Fig. 5A,B). Furthermore, there were no significant differences in *kdrl* expression or enrichment between *ifnphi1^+/+^* and *ifnphi1^−/−^* hearts at 7 dpi (Fig. 5C-E). Using the Tg(kdrl:GFP) reporter we observed a very modest difference between *ifnphi1^+/+^* and *ifnphi1^−/−^* hearts at 7 dpi in the uninjured and injured zones (*P=*0.0463 for uninjured zone and *P=*0.0450 for injured zone, Fig. 5F,G). Given that both uninjured and injured zones showed lower GFP levels in *ifnphi1^−/−^* hearts, together these data suggest that cardiac endothelium regeneration in response to heart injury does not require interferon signaling.

### Interferon signaling is not required for scarless regeneration

**Fig. 6.**
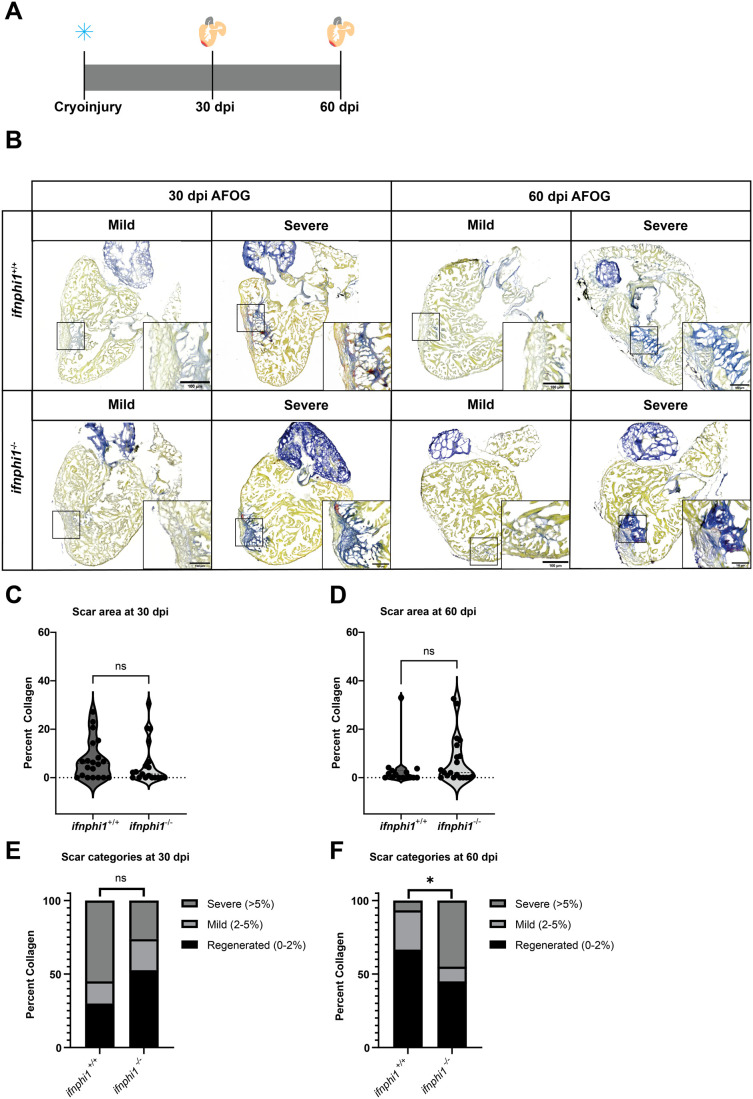
***ifnphi1* mutant hearts are modestly impaired at resolving fibrotic tissue after injury.** (A) Experimental design for heart injury and collection at 30 and 60 dpi. (B) Images of AFOG-stained sections of wild-type and *ifnphi1^−/−^*hearts at 30 and 60 dpi, showing representatives of mild and severe categories of fibrotic tissue resolution. (C,D) Quantification of fibrotic tissue (see Materials and Methods) in AFOG-stained hearts between wild-type and *ifnphi1^−/−^* stained sections at each time point. (E,F) Fibrosis severities for all 30 dpi and 60 dpi injured hearts were categorized into three groups: fully regenerated (0-2% collagen), mildly fibrotic (2-5% collagen), or severely fibrotic (greater than 5% collagen). Chi-squared test for categorical data showed that 30 dpi categories between wild type and *ifnphi1^−/−^* are not significantly different (*P*=0.1517), while 60 dpi categories between wild type and *ifnphi1^−/−^* are modestly statistically significant (*P*=0.0378). The dots on the graph represent the uninjured or injured zones calculated from individual sections of distinct heart ventricles. Data are shown as the mean±s.d.; *n*=19 wild type and *n*=20 *ifnphi1^−/−^* for 30 dpi samples, *n*=15 wild type and *n*=20 *ifnphi1^−/−^* for 60 dpi samples. Scale bars: 50 μm.

Immune signaling is critical for resolution of fibrotic tissue and scarless regeneration. When innate immune cells are depleted or removed from the inflammatory response to heart injury, collagen persists in the wounded area as a thick, irresolvable scar (Lai et al., 2017; Peterson et al., 2024). We investigated whether interferon signaling is necessary for successful heart regeneration at later time points. After heart cryoinjury, adult zebrafish typically resolve their fibrotic tissue between 30 and 60 dpi. We used Acid Fuchsin Orange-G (AFOG) staining to visualize the extent of remaining fibrotic tissue in *ifnphi1^+/+^* and *ifnphi1^−/−^* hearts at 30 and 60 dpi (Fig. 6A,B; Figs S4, S5). We identified the most fibrotic heart section and quantified the size of the remaining fibrotic tissue by normalizing the area of the collagen staining (blue) to the area of the uninjured myocardium (yellow) in each injured heart (Fig. 6C,D). For the 30 dpi samples, the percent collagen in the *ifnphi1^+/+^* and *ifnphi1^−/−^* injured hearts was similar (Fig. 6C), with somewhat more severe injuries in the wild-type hearts. Most hearts had healed from the injury, evidenced by a thicker cortical cardiomyocyte layer and minimal collagen near the injury site. At 60 dpi, the recovery continued in the population of *ifnphi1^+/+^* hearts but stalled in the population of *ifnphi1^−/−^* hearts.

The variation within and between groups at 60 dpi suggested a difference in healing trajectories between the *ifnphi1^+/+^* and *ifnphi1^−/−^* injured hearts. To further investigate these apparent disparities, the injured hearts were categorized based on the percentage of collagen fibrosis as calculated above, representing fully regenerated (0-2% collagen), mildly fibrotic (2-5% collagen), and severely fibrotic (>5% collagen) (Fig. 6E,F). This categorical analysis confirmed that at 30 dpi, more *ifnphi1^+/+^* hearts were severely fibrotic than *ifnphi1^−/−^* hearts, although this difference was not statistically significant (*P*=0.1517). By 60 dpi, 95% of injured *ifnphi1^+/+^* hearts were either fully regenerated or mildly fibrotic, as expected (Fig. 6E,F). However, regeneration in the *ifnphi1^−/−^* hearts did not follow the same progression in recovery. Nearly half of the mutant hearts remained severely fibrotic at 60 dpi. The chi-squared statistical test revealed a modestly significant distinction between *ifnphi1^+/+^* and *ifnphi1^−/−^* heart regeneration at this time point (*P*=0.0378). In summary, our data suggest that interferon signaling has a modest role in resolving fibrotic tissue during zebrafish heart regeneration.

## DISCUSSION

The inflammatory response to zebrafish heart injury clears cellular debris and promotes collagen accumulation and resolution of fibrotic tissue. Our study characterizes the role of type I interferon signaling following cardiac injury in zebrafish, revealing its critical and specific contribution to early inflammation. We found that IFNφ1, produced by the *ifnphi1* gene, is the primary injury-responsive interferon in the heart, and directs upregulation of ISGs. This response is specific to heart injury, and not sham surgery alone, suggesting that *ifnphi1* is activated by DAMPs released from dying cells in the injured myocardium rather than pathogen-associated molecular patterns (PAMPs). This aligns with emerging evidence of non-canonical roles for interferon signaling in tissue repair (Cheng et al., 2008; Lai et al., 2017; Leibowitz et al., 2021). The absence of an endogenous interferon response in injured medaka, which cannot regenerate after heart injury, further highlights its potential importance in creating a pro-regenerative environment (Carey et al., 2024; Lai et al., 2017).

We found that interferon signaling is required to attract neutrophils to the injury site early in zebrafish heart regeneration. While we have referred to this as a defect in ‘neutrophil recruitment’, similar to previous studies (Lai et al., 2017; Goumenaki et al., 2024), it is important to note that we did not distinguish between several possible dynamic cellular processes that may underlie this phenomenon. Defects in chemokine-directed migration, altered retention of circulating cells, or increased movement of neutrophils out of the wounded heart could all result in the observed decrease in neutrophil abundance in the injury site of *ifnphi1*^−/−^ hearts. Live imaging of neutrophil migration following an injury could resolve these mechanistic details in future studies.

Roles for neutrophils have been identified in several tissue regeneration contexts. In mouse skin and spinal cord injuries, when neutrophils were depleted, adult mice exhibited slower wound healing (Nishio et al., 2008; Stirling et al., 2009). Zebrafish neutrophils control epicardial cell expansion just days after heart injury (Peterson et al., 2024). When zebrafish neutrophils were depleted, there was significantly less epicardial cell expansion and less cardiomyocyte proliferation; however, the long-term effects of neutrophil depletion were not studied. Here, we found a correlation between interferon signaling, neutrophil recruitment, and timely resolution of fibrotic tissue during zebrafish heart regeneration.

We also found that disrupting interferon signaling did not impact macrophage recruitment to the site of injury or interfere with the re-growth of new blood vessels. We suspect that other pro-inflammatory cytokines, independent of the interferon signaling pathway, were likely sufficient to recruit macrophages and trigger vascular growth in the injured myocardium (Luz et al., 2025; Nguyen-Chi et al., 2017). Interestingly, the artificial induction of interferon signaling in the medaka heart, via Toll-like receptor activation, enhanced macrophage recruitment, not neutrophil recruitment (Lai et al., 2017). This highlights the differences in immune response to injury between species. Notably, a recent preprint, posted while our study was under review, found that interferon-gamma signaling is required for macrophage-mediated debris clearance after heart injury (Lim et al., 2026 preprint), raising the exciting possibility that multiple interferon signaling pathways are integrated during zebrafish heart regeneration.

We found a mild regeneration disparity between the *ifnphi1^+/+^* and *ifnphi1^−/ −^* injured hearts, where regeneration stalls in the absence of interferon signaling. We hypothesize that the early impact on neutrophil recruitment in *ifnphi1^−/−^* hearts may have resulted in slower wound healing. Elucidating the specific mechanisms that connect neutrophil activity with resolution of fibrotic tissue remains an exciting avenue for study. Future work could interrogate these mechanisms using mutants or small-molecule inhibitors of neutrophil activity (Isles et al., 2019; Kell et al., 2018; Peterson et al., 2024). Despite reduced neutrophil infiltration in all mutant hearts, approximately half of *ifnphi1^−/−^* fish were still able to regenerate completely by 60 dpi. This suggests that the inflammatory process, including phagocytosis of cellular debris and remodeling of the extracellular matrix to facilitate regenerative resolution of fibrotic tissue, was adequately executed in the absence of interferon signaling in these individuals. This variability between individuals may have many underlying reasons, including variation in the intensity of injury or more subtle differences in the tempo of damage sensing, cell recruitment, or tissue regrowth. Future studies that connect the initial responses to interferon signaling to neovascularization, collagen deposition, and cardiomyocyte proliferation and dedifferentiation will enable mechanistic connections between interferon signaling and these later processes in zebrafish heart regeneration.

Collectively, this study underscores the concept of regenerative robustness, where redundant mechanisms protect outcomes to the function of the organ and organism. Understanding how these mechanisms drive zebrafish heart regeneration may reveal novel therapeutic targets to promote repair in non-regenerative mammalian hearts, where key compensatory pathways may be lost or insufficient.

## MATERIALS AND METHODS

### Fish husbandry and zebrafish lines

All zebrafish lines were maintained in accordance with standard husbandry practices at the CBRZ zebrafish facility at the University of Utah. This study was conducted with approval of the Office of Institutional Animal Care and Use Committee (IACUC no. 20-07013) of the University of Utah's animal care and use program.

### Generation of a zebrafish *ifnphi1* mutant line

To generate a stable *ifnphi1* mutant line, *ifnphi1* was targeted using CRISPR-Cas9 gene editing. Four guide RNAs (gRNAs) targeting exons 1-3 of the *ifnphi1* gene were designed and ordered from IDT. To generate the crRNA/gRNA duplex for each gRNA, a 100 µM solution of crRNA and tracrRNA was made for each duplex in IDT gRNA buffer. Equal volume of tracrRNA (5 μl) and gRNA (5 μl) was combined annealed via PCR to generate the crRNA-gRNA duplex for each gRNA. The PCR program to anneal crRNA and gRNA is as follows: 95°C, 5 min; cool at 0.1°C/s to 25°C; 25°C, 5 min; cool to 4°C rapidly.

Along with these gRNAs (800 ng/μl), SpCas9 protein (from IDT), and Phenol Red were co-injected into WT embryos. Total volumes: crRNA/gRNA 1-4 (1.24 μl each), SpCas9 (0.5 μl at 10 μg/μl), and Phenol Red (1 μl). 1 nl was injected into each embryo. Mosaic embryos were raised to adulthood and incrossed with each other before being outcrossed to wild-type fish. Primers amplifying the CRISPR-editing region were used to select outcrossed fish with large deletions. Specifically, embryos from this outcross carry large deletions, ∼800 bp. To establish a homozygous mutant line, these heterozygous mutant fish were incrossed. Three primers were used to distinguish between homozygous mutants, heterozygous fish, and wild-type fish in two separate PCR reactions (Table S1). All experiments presented compare homozygous mutant zebrafish with WT siblings. This line was given the designation *ifnphi^zj7^*. Mutant zebrafish were crossed into the Tg(mpx:GFP) line to label neutrophils (Buchan et al., 2019).

### Cardiac cryoinjury

Cryoinjuries were performed on the ventricular apex of anesthetized zebrafish as described previously (González-Rosa et al., 2011). 0.02% Tricaine (MS-222) was used to anesthetize zebrafish. Zebrafish were positioned on a moist sponge. A small thoracic incision was made with forceps and dissecting scissors to expose the ventricular apex. Using a liquid nitrogen-chilled copper wire cryoprobe (0.5 mm in diameter, as previously described; González-Rosa and Mercader, 2012), the ventricular apex was injured with the probe for 23 s. Following injury, the fish were revived in freshwater tanks and returned to the facility for monitoring. Both male and female adult zebrafish between 6 and 12 months of age were studied, in roughly equal proportions although sex was not tracked.

### Histological methods

Hearts were fixed in 4% paraformaldehyde (PFA, Electron Microscopy Sciences) in 1X PBS for 24 h, then sucrose-treated sequentially from 10% to 30% sucrose. Sucrose-treated hearts were then mounted in Optimal Cutting Temperature (OCT, Fisher HealthCare) embedding medium and frozen at −80°C. Hearts from Tg(mpx:GFP) and Tg(kdrl:GFP) animals were sectioned at 12 μm and mounted in Fluoroshield with DAPI (Sigma-Aldrich). For AFOG (Sigma-Aldrich) staining, OCT-mounted hearts were sectioned at 7 μm. Bouin's solution (Sigma-Aldrich) was used to fix heart tissue before staining with PT/PM (phosphotungstic acid/phosphomolybdic acid, Sigma-Aldrich) and AFOG stain. After staining, sections were washed twice with 0.5% acetic acid (30 s per wash), four times with 100% ethanol (1 min per wash), and three times with xylenes (5 min per wash). For AFOG imaging, the ECHO Revolution 2.0 confocal microscope was used to capture brightfield images at 10X.

### RNAscope *in situ* hybridization

Zebrafish hearts were fixed for 24 h in 4% paraformaldehyde (PFA) in 1X PBS, sequentially sucrose-treated (10% to 30%), and OCT embedded. Hearts were then cryosectioned at 12 μm. RNAscope (Advanced Cell Diagnostics, Hayward, CA, USA) was performed using the RNAscope^®^ Multiplex Fluorescent Detection Kit v2 protocol, with previously published probes (Carey et al., 2024). The Zeiss 880 confocal microscope was used to capture RNAscope images at 40X. While RNAscope is a commonly used method for RNA-FISH, it should be acknowledged that the specificity of the probes used in these experiments have not been explicitly validated.

### Imaging quantification

ImageJ was used to analyze images of heart sections probed for *myl7* (cardiomyocytes), *mpeg1.1* (macrophages), *isg15* (interferon-stimulated gene-15), *kdrl* (vascular), and DAPI. To separate the wound from the uninjured ventricle, we set a threshold on the cardiomyocyte channel to create an uninjured ventricle mask. Using the DAPI channel and the difference between the uninjured ventricle mask and the DAPI mask, an injured area mask was created for every heart. Masked images of each probe in the injured and uninjured areas were uploaded to CellProfiler software (version 4.2.1) using a modified CellProfiler ‘Speckle Counting’ pipeline (Stirling et al., 2021). Total number of nucleated cells in the DAPI channel and *mpeg1.1*, *isg15*, and *kdrl* speckles were identified using the IdentifyPrimaryObjects module. Macrophages and nuclei were related using the RelateObjects CellProfiler module. The percent of macrophages relative to the total number of cells in injured and uninjured areas was calculated. Both *isg15* and *kdrl* speckles were counted regardless of the nuclei relationship. Percent *kdrl* and *isg15* speckles relative to the total number of cells in injured and uninjured areas was calculated. Furthermore, the enrichment of *kdrl* expression in the injured versus uninjured section was calculated for each heart.

For Tg(kdrl:GFP) analysis, the percent of kdrl pixels was calculated as an area fraction of the uninjured or injured areas of each heart using ImageJ.

For Tg(mpx:GFP) neutrophil counts in injured wildtype and *ifnphi1^−/−^* fish at 1 and 3 dpi, uninjured and injured masks were created using ImageJ as described above. A similar CellProfiler ‘Speckle Counting’ pipeline was used to count the number of neutrophils in the injured and uninjured areas.

For fibrotic tissue quantification in AFOG images, ImageJ software was used. Here, the fibrotic tissue size was calculated by measuring the collagen (blue) stained area and divided by the total myocardium and multiplied by 100 to generate a percentage. All animals in these experiments were derived from a large cohort of animals injured across several successive days. We typically injured ∼20 hearts from both genotypes per day and carried out injuries across multiple days to accumulate enough fish for the entire timecourse. Scarred scoring was performed blind.

### RT-qPCR

For experiments that isolated injured and uninjured areas of the heart, the injured ventricles were first isolated from the bulbus arteriosus and atrium. Then, for each injured heart, the injured zone was isolated from the uninjured zone using dissection scissors. Three injured zones (*n*=3 hearts) or three uninjured zones (*n*=3 hearts) were pooled together per RNA extraction reaction. RNA was purified from injured ventricles using PicoPure (Thermo Fisher Scientific) and converted to complementary DNA (cDNA) using QuantiTect Reverse Transcription Kit, using the included RT primer mix (Qiagen). cDNA was amplified using BrilliantII SYBR Green QPCR Master Mix (Agilent) with 10 pmol of each primer. Quantitative reverse-transcription PCR (qRT-PCR) was performed using the QuantStudio 5 (Thermo Fisher Scientific) protocol. Elongation factor 1-alpha (*ef1α*) expression was measured for each biological triplicate in every experiment. The ΔCt method was used to analyze the data, with *ef1α* as the normalization factor for each sample. The fold change was calculated using the ΔΔCt method, where 2−ΔΔCt of injured samples is relative to the average of 2−ΔΔCt of uninjured heart samples.

### GFP:Boost antibody staining

Tg(kdrl:GFP) sections 12 μm thick were first washed in 1XPBS three times. Slides were then permeabilized with 0.3% Triton in 1XPBS for 10 min and then washed again in 1XPBS twice. Then, incubated with block in PBS plus 5% heat inactivated goat serum, 3% BSA, and 0.1% Triton for 1 h. Slides were then incubated overnight with 1:500 GFP:Boost (Chromotek, GFP:Booster ATTO 488). Finally, slides were washed with 1XPBS three times and mounted in Fluoroshield with DAPI (Sigma-Aldrich). The Zeiss 880 confocal microscope was used to capture RNAscope images at 63X.

### Statistical analysis

Statistical analyses for column and nested data were performed using GraphPad Prism (v.10.5.0). Data distribution was assessed using the Shapiro-Wilk normality test. The significance level was set to *P*=0.05, and the standard deviation from the mean is indicated on the figures. To determine the statistical significance of the normal data, Welch's *t*-test was used.

For the categorical fibrotic tissue analysis (Fig. 6E,F), a chi-squared test statistic was calculated for both the 30 dpi and 60 dpi datasets. Then, using this chi-squared test statistic and degrees of freedom=2, a *P*-value was derived.

## Supplementary Material



10.1242/biolopen.062374_sup1 Supplementary information

Table S1. Sequence information for *ifnphi1*, *ifnphi1* mutant genotyping, RT-qPCR primer sequences, and gRNA design.
