# Supplementary material for: Interferon signaling is required for early neutrophil recruitment after zebrafish heart injury
Source: Biol Open. 2026 Jun 15;15(6):bio062374. doi: 10.1242/bio.062374 (PMC13312930; doi:10.1242/bio.062374)
Supplement: Supplementary information [file biolopen-15-062374-s1.pdf]

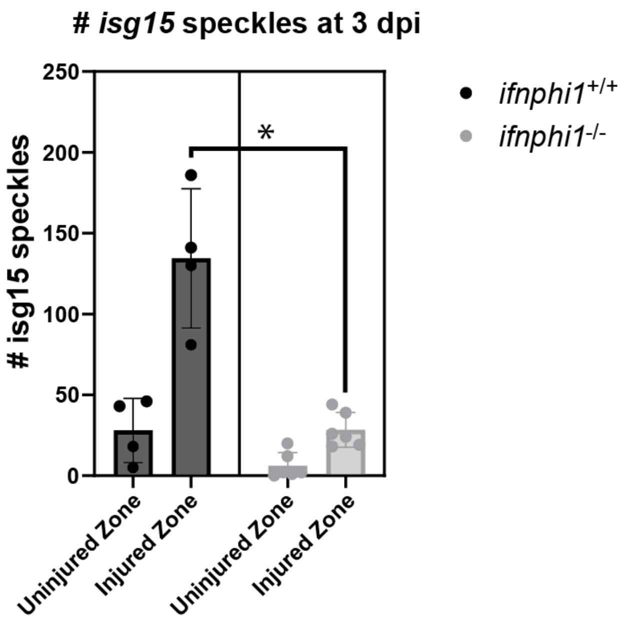

Fig. S1. *isg15* speckle count in *ifnphi1*$^{-/-}$ versus wild-type injured ventricles at three dpi. The number of *isg15* speckles counted using the CellProfiler pipeline is represented in the injured and uninjured zones of each fish at 3 dpi. Each dot represents the injured and uninjured zones of one ventricle. Differences in *isg15* expression in the injured zones between *ifnphi1*$^{-/-}$ and wild-type were significant with p=0.0141 using Welch's t-test.

Biology Open • Supplementary information

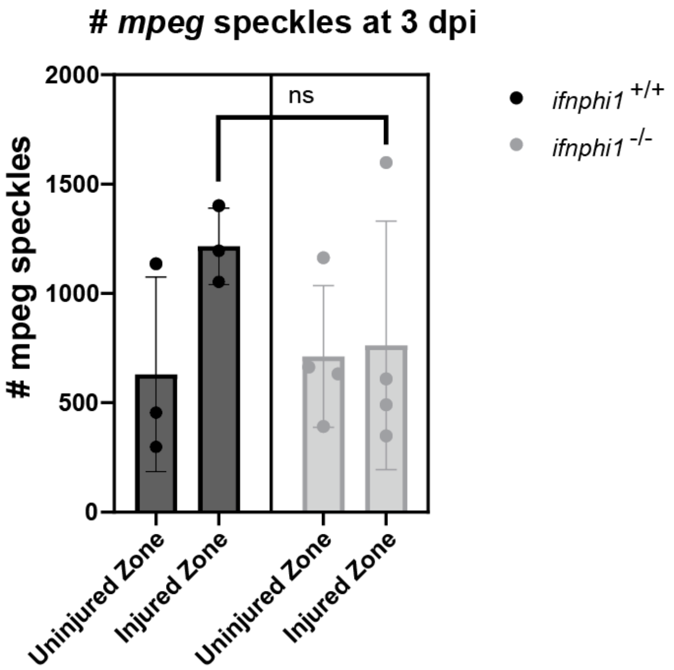

Fig. S2. *mpeg* speckle count in *ifnphi1*$^{-/-}$ versus wild-type injured ventricles at three dpi. The number of *mpeg* speckles counted using the CellProfiler pipeline is represented in the injured and uninjured zones of each fish at 3 dpi. Each dot represents the injured and uninjured zones of one ventricle. Differences in *mpeg* expression in the injured zones between *ifnphi1*$^{-/-}$ and wild-type were not significant with p=0.2119 using Welch's t-test.

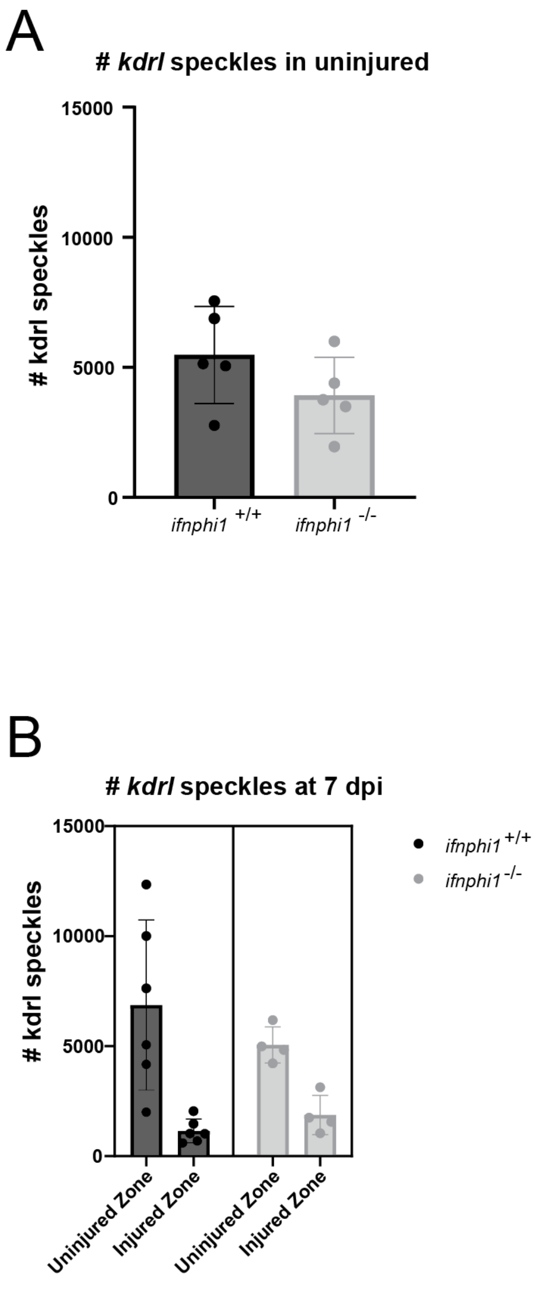

Fig. S3. *kdrl* speckle count in *ifnphi1$^{-/-}$* versus wild-type injured ventricles. A,B) The number of *kdrl* speckles counted using the CellProfiler pipeline is represented in the injured and uninjured zones of each fish at 7 dpi. Each dot represents the injured and uninjured zones of one ventricle. Differences in *kdrl* expression in the injured zones between *ifnphi1$^{-/-}$* and wild-type were not significant at any post-injury time point.

Biology Open • Supplementary information

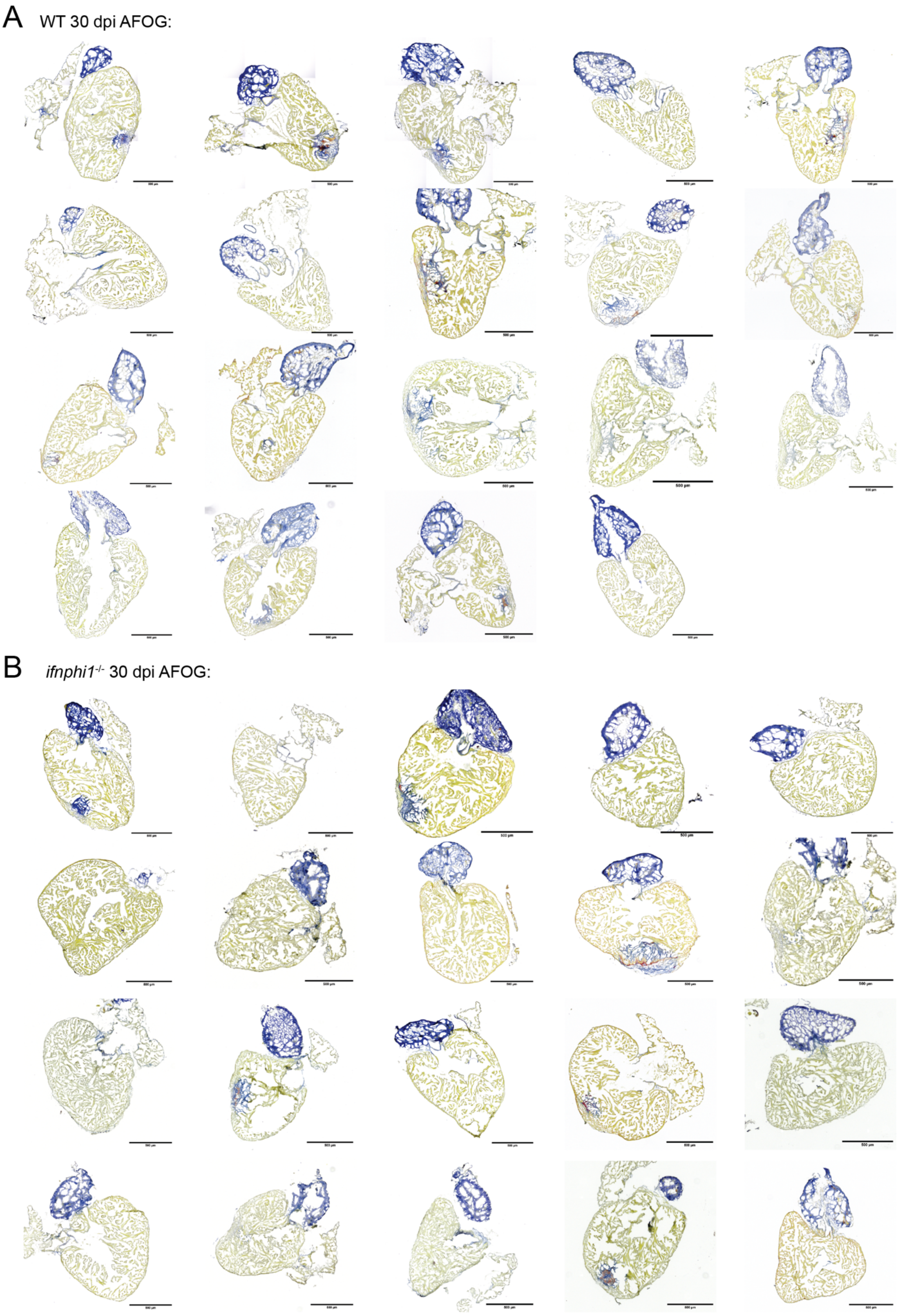

Fig. S4. All AFOG images at 30 dpi. A) All wild-type hearts 30 dpi AFOG stained. B) All *ifnphi1*$^{-/-}$ hearts 30 dpi AFOG stained. Scale bars = 500 µm.

Biology Open • Supplementary information

A WT 60 dpi AFOG:

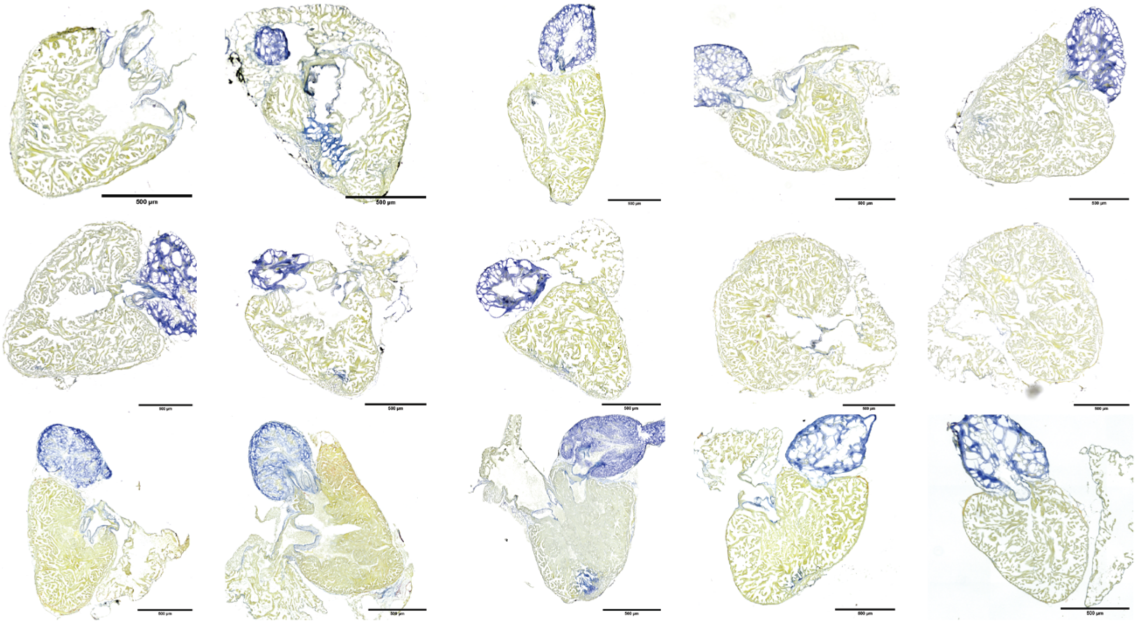

B *ifnphi1*$^{-/-}$ 60 dpi AFOG:

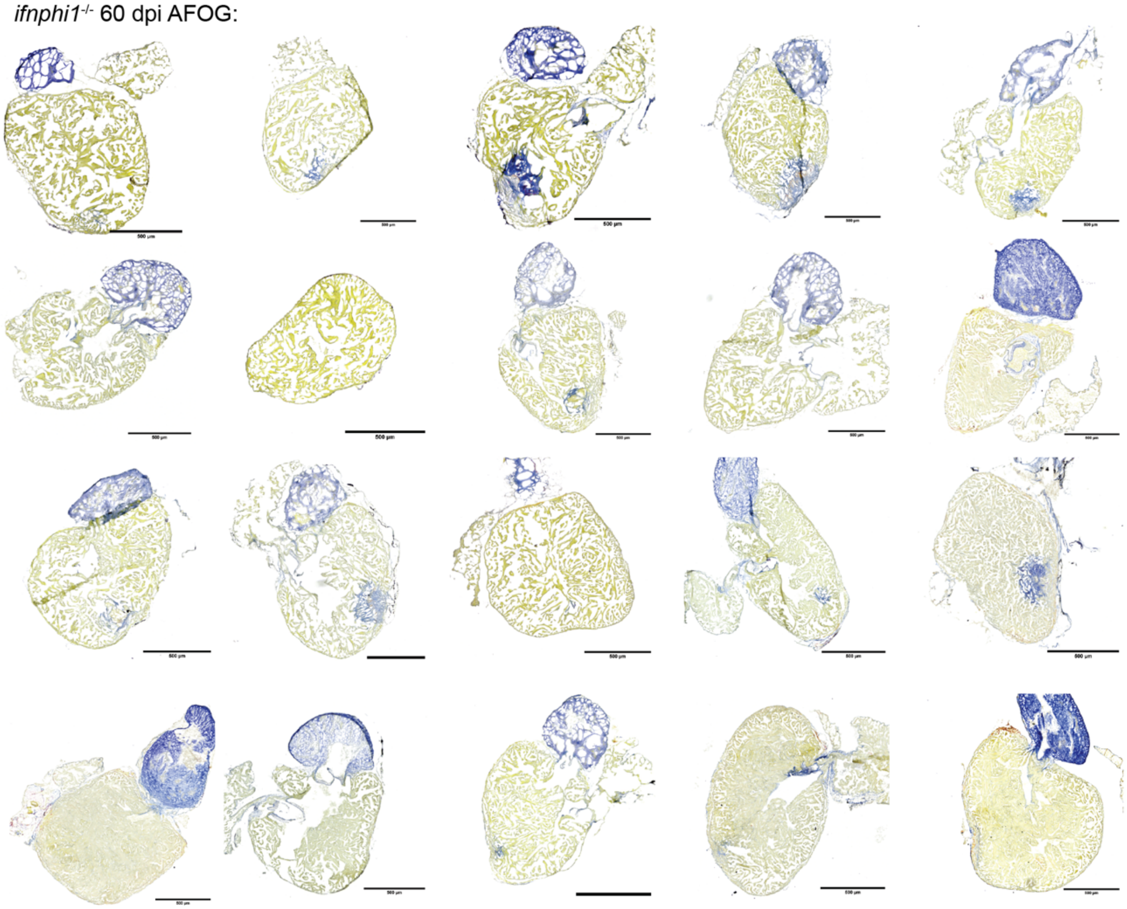

Fig. S5. All AFOG images at 60 dpi. A) All wild-type hearts 60 dpi AFOG stained. B) All *ifnphi1*$^{-/-}$ hearts 60 dpi AFOG stained. Scale bars = 500 µm.

Table S1. Sequence information for *ifnphi1*, *ifnphi1* mutant genotyping, RT-qPCR primer sequences, and gRNA design.

Available for download at
https://journals.biologists.com/bio/article-lookup/doi/10.1242/bio.062374#supplementary-data
