# Supplementary material for: Interferon signaling is required for early neutrophil recruitment after zebrafish heart injury
Source: Biol Open. 2026 Jun 15;15(6):bio062374. doi: 10.1242/bio.062374 (PMC13312930; doi:10.1242/bio.062374)
Supplement: Supplementary file 1 [file biolopen-15-062374_review_history.pdf]

Interferon signaling is required for early neutrophil recruitment after zebrafish heart injury

Alexis V. Schmid and James Gagnon

10.1242/bio.062374

Editor: Daniel Gorelick

Review timeline

Original submission:	16 November 2025
Editorial decision:	23 November 2025
First revision received:	13 April 2026
Editorial decision:	24 April 2026
Second revision received:	5 May 2026
Accepted:	8 May 2026

Original submission

First decision letter

MS ID#: bio.062374

MS Title: Interferon signaling is required for early neutrophil recruitment after zebrafish heart injury

Authors: Alexis V. Schmid and James Gagnon

Thank you for submitting your manuscript to Biology Open. Your manuscript was reviewed as part of our Fast & Fair peer review initiative, where we deliver decisions, with reviews, within 7 working days of submission. If you have any comments on this experience, please let us know! For more on this initiative, see https://journals.biologists.com/bio/pages/fast-fair

I have now reached a decision on the above manuscript. The reviewer reports are shown at the bottom of this email.

As you will see, the reviewers raised a number of substantial criticisms that prevent me from accepting the paper at this stage.

They suggest, however, that a revised version might prove acceptable, if you can address their concerns. If you think that you can deal satisfactorily with the criticisms on revision, I would be pleased to see a revised manuscript. We would then return it to the reviewers.

At this stage, we also ask you to ensure your manuscript complies with our formatting guidelines. Provided you are able to fully address the referees' comments, we are positive about publication of your paper (we accept over 95% of revision submissions) and therefore hope you won't mind any extra work involved in reformatting your manuscript at this point.

Please upload both a 'clean' version of your Word file, along with a highlighted version clearly showing where you have made changes in the revised manuscript. Please avoid using 'Track changes' in Word files as these are lost in PDF conversion.

© 2026. Published by The Company of Biologists under the terms of the Creative Commons Attribution License (https://creativecommons.org/licenses/by/4.0/).

I should be grateful if you would also provide a point-by-point response detailing how you have dealt with the points raised by the reviewers in the 'Response to Reviewers' box. Please attend to all of the reviewers' comments. If you do not agree with any of their criticisms or suggestions please explain clearly why this is so.

Reviewer 1

Comments for the author

In this study, Schmid and Gagnon report that interferon signaling acts as a regulator of neutrophil behavior during heart regeneration in zebrafish. The authors identify a transient early upregulation of ifnphi1 following ventricular cryoinjury. To investigate the role of interferon signaling in a regeneration-competent model, they generated an ifnphi1 mutant line. These mutants exhibit reduced numbers of neutrophils at early post-injury stages, while showing no apparent defects in macrophage numbers or revascularization.
To evaluate potential impairments in fibrosis resolution, the authors analyzed injured hearts at later time points and observed increased collagen deposition in mutant ventricles. Together, these findings implicate interferon signaling in regulating the early neutrophil response to injury and suggest that perturbations in neutrophil dynamics influence fibrosis resolution.

Major comments:
- The low-magnification ventricular sections presented throughout the manuscript are difficult to interpret. The section plane and injury size need to be comparable across experiments and consistent with standards in the field. Likewise, injured areas should be clearly delineated, and image resolution should be sufficiently high to allow proper assessment. It is unclear whether this issue stems from file compression, but many ventricular section images appear pale and low in resolution.
- Increase sample number. In some experiments, only two biological replicates are presented per condition, which is insufficient. Even in cases where n = 3, this may not provide adequate statistical power, particularly given the variability observed across phenotypes and gene-expression measurements.

- Figure 1. It is unclear whether statistical analyses were performed. Given the variability in expression, qPCR analyses using RNA isolated specifically from the injured region rather than from whole ventricles would be more informative. This is especially important for the 2 hpi time point in Fig. 1A,B and the 72 hpi time point in Fig. 1D.
- Where is ifnphi1 expressed during homeostasis and after injury.?
- Figure 2B. isg15 appears to be expressed early after injury in ifnphi1 mutants, whereas it is not detectable from 2-24 hpi in wild types (Fig. 1D). Additionally, ifnphi1 induction at 2 hpi is comparable between mutants (Fig. 2E) and wild types (Fig. 1B). Given these observations and the nature of the mutation, is it possible that the ifnphi1 mutant is not a full knockout but a hypomorphic allele? Also, the authors should address how these data can be explained.
- The lack of isg15 expression at 3 dpi is interesting. Where is isg15 expressed? Colocalization with different markers (ECs, macrophages, neutrophils, EPDCs) would greatly help to interpret the data.
- Interpretation of Figure 3: The authors state that interferon signaling promotes neutrophil recruitment; however, no data in the manuscript directly support this conclusion. Gain-of-function experiments would be required, and recruitment is inherently dynamic, requiring live imaging. Please correct the text (and title) accordingly or provide data to support these claims.
- Figure 4. Although mpeg is used as a macrophage marker, it would be important to show that this probe does not mark neutrophils or other cells in the injury. This is of special importance as based on the images provided reliable quantification seems extremely challenging. Colocalization analyses using a neutrophil marker (e.g., Tg(mpx:GFP)) should be performed.
- Figure 5. RNA-FISH for kdrl is not appropriate to assess neovascularization. kdrl is expressed in endocardial and vascular endothelial cells as well as in CMs (at low levels). To evaluate vascular defects, the authors should use vascular reporters and/or antibody staining for appropriate established markers (e.g., Fli, Aldh1a2, etc).
- It would be informative to examine whether mutants display defects in CM proliferation or dedifferentiation, which could contribute to the reduced regenerative capacity observed.
- Similarly, it would be interesting to assess whether collagens are upregulated in injured mutants compared with wild types.

© 2026. Published by The Company of Biologists under the terms of the Creative Commons Attribution License (https://creativecommons.org/licenses/by/4.0/).

Minor comments:
- Figure 1C. use "hps" (hours post sham) rather than "hpi"
- HPI and DPI are used throughout the manuscript, please standardize.
- The term "scar" implies permanence and should not be used for zebrafish, where fibrosis is transient. Instead, terms such as "fibrosis," "collagen accumulation," or "fibrotic tissue" are appropriate.
- Line 15: Please clarify the meaning of "fresh" CMs?
- Line 96: Please clarify what "distantly related" means here.

Reviewer 2

Comments for the author

The manuscript by Schmid and Gagnon addresses an interesting question in cardiac regeneration, focusing on how type I interferon signalling, specifically IFNϕ1, shapes the early inflammatory response and later regenerative outcome upon zebrafish cardiac injury. The work is well executed and overall the data support a role for IFNϕ1 in driving an interferon-stimulated gene programme and in promoting early neutrophil accumulation in the injured heart. However, a few additional controls, clarifications and/or some softening of language is need to make sure the study makes a solid mechanistic contribution to how early innate signalling regulates zebrafish heart regeneration- please see points below.
1. The study relies entirely on the CRISPR ifnphi1 deletion but does not directly demonstrate loss or strong reduction of ifnphi1 transcript, as shown in Figure 2E. An explanation to the observed lack of effect on ifnphi1 transcript levels should be included and the use or an orthogonal method such as HCR/RNAscope with probes designed against the mutated transcripts would be necessary to directly validate the mutant specificity.
2. The ifnphi1 mutant data are interpreted as a defect in neutrophil recruitment to the injury, but the description does not make it clear whether total neutrophil numbers per ventricle (injured + uninjured) differ between mutant and wild type counterparts, nor whether neutrophil density in uninjured myocardium is unchanged. Without these controls, the phenotype could equally represent reduced myelopoiesis or survival rather than altered recruitment.
3. In the comparison of scar resolution at 30 dpi and 60 dpi it will be important to clarify whether these hearts derive from the same injury cohort.
4. For all RNAscope figures, including supplementary panels, scale bars and injury borders must be clearly shown on the representative images. This would improve readability/clarity on the spatial distribution of signal relative to the wound and the regions used for quantification.
5. The Methods should specify the sex and approximate age range of the zebrafish used for injury experiments, whether fish were randomised to treatment and if injuries and scar scoring were performed blind to genotype.

Reviewer's Responses to Questions

Experimental quality
Does each figure have the proper controls?
If 'No', please indicate reasons in Comments for Author box below.
Reviewer #1:
- Yes

Reviewer #2:
- No

Were the data analyzed using appropriate statistical tests?
If 'No', please indicate reasons in Comments for Author box below.
Reviewer #1:
- Yes

Reviewer #2:

© 2026. Published by The Company of Biologists under the terms of the Creative Commons Attribution License (https://creativecommons.org/licenses/by/4.0/).

- Yes

Reproducibility
Were experiments performed using adequate number of biological replicates?
If 'No', please indicate reasons in Comments for Author box below.
Reviewer #1:
- No

Reviewer #2:
- Yes

Does the methods section provide sufficient detail to permit reproducibility?
If 'No', please indicate reasons in Comments for Author box below.
Reviewer #1:
- Yes

Reviewer #2:
- Yes

Completeness
Are the manuscript's conclusions supported by the data?
If 'No', please indicate reasons in Comments for Author box below.
Reviewer #1:
- Yes

Reviewer #2:
- Yes

Scholarship
Do the authors cite and discuss the merits of data that would argue for and against their conclusion?
If 'No', please indicate reasons in Comments for Author box below.
Reviewer #1:
- Yes

Reviewer #2:
- Yes

Does the manuscript title & abstract accurately reflect the contents of the manuscript, without hyperbole?
If 'No', please indicate reasons in Comments for Author box below.
Reviewer #1:
- No

Reviewer #2:
- Yes

First revision

Author response to reviewers' comments

© 2026. Published by The Company of Biologists under the terms of the Creative Commons Attribution License (https://creativecommons.org/licenses/by/4.0/).

Reviewer 1: In this study, Schmid and Gagnon report that interferon signaling acts as a regulator of neutrophil behavior during heart regeneration in zebrafish. The authors identify a transient early upregulation of ifnphi1 following ventricular cryoinjury. To investigate the role of interferon signaling in a regeneration-competent model, they generated an ifnphi1 mutant line. These mutants exhibit reduced numbers of neutrophils at early post-injury stages, while showing no apparent defects in macrophage numbers or revascularization. To evaluate potential impairments in fibrosis resolution, the authors analyzed injured hearts at later time points and observed increased collagen deposition in mutant ventricles. Together, these findings implicate interferon signaling in regulating the early neutrophil response to injury and suggest that perturbations in neutrophil dynamics influence fibrosis resolution.

We thank this reviewer for their careful reading of our manuscript and their constructive criticism. We have revised based on their comments - including new experiments and analysis - and now present an improved manuscript. A point-by-point response is below.

Major comments:
- The low-magnification ventricular sections presented throughout the manuscript are difficult to interpret. The section plane and injury size need to be comparable across experiments and consistent with standards in the field. Likewise, injured areas should be clearly delineated, and image resolution should be sufficiently high to allow proper assessment. It is unclear whether this issue stems from file compression, but many ventricular section images appear pale and low in resolution.

This was mainly an issue with image compression during PDF conversion and has been remedied in this revised manuscript. The injury size, section thickness, and section plane were calibrated following standard protocols described in many manuscripts from our field. As with many studies, there is minor variation in heart orientation during mounting that impacts the section plane relative to the injury site. All heart sections for fluorescence microscopy were imaged with a 40X objective, while AFOG stained sections were cut thinner and imaged with a 10X objective. All of these parameters were adopted following standards from the zebrafish heart literature and detailed in the Methods.

- Increase sample number. In some experiments, only two biological replicates are presented per condition, which is insufficient. Even in cases where n = 3, this may not provide adequate statistical power, particularly given the variability observed across phenotypes and gene-expression measurements.

We have repeated many experiments with additional biological replicates. As discussed below, we also dissected the injured zone vs. uninjured zone from cryoinjured hearts. These data are now presented in Figures 1 and 2. As this reviewer noted, these snapshot measurements of a process with inherent temporal variation can lead to variability, but the increased replicates permitted statistical testing to evaluate and support our conclusions.

- Figure 1. It is unclear whether statistical analyses were performed. Given the variability in expression, qPCR analyses using RNA isolated specifically from the injured region rather than from whole ventricles would be more informative. This is especially important for the 2 hpi time point in Fig. 1A,B and the 72 hpi time point in Fig. 1D.

This is an excellent suggestion. We repeated these experiments, now dissecting the injured zone and uninjured zone from cryoinjured hearts. These data are now presented in Figures 1 and 2. These data reinforce our conclusion that ifnphi1, and not other ifnphi genes, is expressed in the injured zone of cryoinjured hearts.

- Where is ifnphi1 expressed during homeostasis and after injury.?

In our previous study (Carey et al., *Biology Open* 2024), we reported that ifnphi1 is not expressed in the uninjured zebrafish heart, and is expressed after cryoinjury in endothelial cells. We have not repeated this single-cell RNAseq experiment at early time points immediately after injury. However, this previously-published induction of ifnphi1 after injury is consistent with our RT-qPCR data presented in this manuscript. Given the qPCR data we now

© 2026. Published by The Company of Biologists under the terms of the Creative Commons Attribution License (https://creativecommons.org/licenses/by/4.0/).

present in Figure 1, we conclude that ifnphi1 is expressed in the injury zone 2 hours after cryoinjury, likely in endothelial cells. We revise the text on line 181 to make this clear.

Our attempts to use RNAscope to image ifnphi1 expression in injured hearts have failed - we were warned by ACDBio that the short length of these transcripts (the gene is very small) made probe design challenging, and in our hands these probes gave no signal, even in positive control samples from other tissues.

- Figure 2B. isg15 appears to be expressed early after injury in ifnphi1 mutants, whereas it is not detectable from 2-24 hpi in wild types (Fig. 1D). Additionally, ifnphi1 induction at 2 hpi is comparable between mutants (Fig. 2E) and wild types (Fig. 1B). Given these observations and the nature of the mutation, is it possible that the ifnphi1 mutant is not a full knockout but a hypomorphic allele? Also, the authors should address how these data can be explained.

We thank the reviewer for their careful reading and comparison of our data presented across multiple panels. As mentioned above, we have now added additional biological replicates, and included dissected uninjured zone and injured zone from wild-type and mutant cryoinjured hearts. We have also simplified the way we present these data to avoid confusion. Therefore, these panels in Figures 1 and 2 have now changed, but our conclusions remain the same and are now supported by statistical tests. Notably - from our qPCR data, we conclude that isg15 is only expressed in the injured zone of wild-type cryoinjured hearts at 3 dpi (Figure 1D), and not expressed in the uninjured zone of wild-type hearts (Figure 1D), or any part of ifnphi1-/- mutant hearts (Figure 2B). This conclusion is supported by the RNAscope data in Figure 2C, quantified in Figure 2D.

We expected that ifnphi1 expression would be similar in cryoinjured wild-type and mutant hearts. Our zj7 mutant allele of ifnphi1 is a large in-frame deletion, which should not induce NMD-mediated degradation of the mRNA. To detect expression of wild-type and ifnphi1 mutant mRNA, we deliberately used qPCR primers that span exons 4-5, outside the zj7 deletion, to measure mRNA expression levels. Expression is quite low and variable, but this is why it should be similar across genotypes.

Several lines of evidence suggest that the zj7 allele is a null mutant. First, as previously mentioned, there is no isg15 expression after heart injury in ifnphi1 mutants (Figure 1B). Second, AlphaFold prediction of the ifnphi1 protein structure suggests that this mutation will destroy the correct fold of the protein - it deletes >30% of amino acids from the mature ifnphi1 protein sequence, eliminating or dramatically altering 3 of the 6 alpha helices that form the barrel-like central structure of this signaling protein. This can be visualized on the AlphaFold predicted structure of D. rerio ifnphi1 protein at this link https://alphafoldserver.com/fold/42d87d06e8078c20, where amino acids 30 - 78 are deleted in the mutant allele. We attempted to confirm that this mutant protein was non-functional by cloning wild-type and zj7 mutant cDNAs and injecting them into a isg15:GFP transgenic line that we have previously generated (Balla et al., *Current Biology* 2020). Unfortunately these tests were inconclusive - neither wild-type or mutant ifnphi1 was capable of activating the reporter in the early stage embryo, suggesting that the interferon signaling pathway is not present in the early embryo. This is consistent with the absence of interferon receptor expression in published zebrafish embryo datasets like Daniocell (Sur et al., *Dev. Cell* 2023). Still, these data together suggest that our zj7 ifnphi1 mutant allele is a true null allele.

- The lack of isg15 expression at 3 dpi is interesting. Where is isg15 expressed? Colocalization with different markers (ECs, macrophages, neutrophils, EPDCs) would greatly help to interpret the data.

In our previous study (Carey et al., *Biology Open* 2024), we reported that isg15 (and other genes induced by interferon signaling) is expressed after cryoinjury in endothelial, epicardial and leukocyte cell types. We now revise the text to make this prior knowledge clear.

- Interpretation of Figure 3: The authors state that interferon signaling promotes neutrophil recruitment; however, no data in the manuscript directly support this conclusion. Gain-of-function experiments would be required, and recruitment is inherently dynamic, requiring live imaging. Please correct the text (and title) accordingly or provide data to support these claims.

© 2026. Published by The Company of Biologists under the terms of the Creative Commons Attribution License (https://creativecommons.org/licenses/by/4.0/).

We appreciate the opportunity to clarify what we mean with the term 'recruitment.' Previous publications have shown that few neutrophils are present in the uninjured homeostatic zebrafish heart. After cryoinjury, neutrophils enter the heart and accumulate at the site of injury. We now quantify the total number of neutrophils in wild-type and ifnphi1 mutant hearts and find that there are less neutrophils in injured ifnphi1 mutant hearts at both 1 and 3 days post-injury (Figure 3C and 3F). Normalized to the area of uninjured and injured zones, we find that this decreased recruitment is specific to the injured zone at both timepoints in ifnphi1 mutant hearts (Figure 3B and 3E). We deliberately chose the term recruitment to refer to this increase in the number of neutrophils in the heart overall and to the site of injury. Given that there are very few neutrophils in the heart before injury, and after injury they are found in the heart and at highest concentration within the injured zone, we feel comfortable implying a deliberate localization of neutrophils to the wound. Together with previous Figures, we suggest that this recruitment is driven at least in part by interferon signaling. Live imaging would be a great way to confirm this hypothesis by tracking live neutrophils and their directional movement, but ex vivo imaging of an intact heart for days after cryoinjury is extremely challenging and outside the scope of this study.

- Figure 4. Although mpeg is used as a macrophage marker, it would be important to show that this probe does not mark neutrophils or other cells in the injury. This is of special importance as based on the images provided reliable quantification seems extremely challenging. Colocalization analyses using a neutrophil marker (e.g., Tg(mpx:GFP)) should be performed.

Unfortunately, crossing our ifnphi1 mutant into many, many transgenic backgrounds to confirm every RNAscope probe would be both time-consuming and costly. We have done so for several transgenic lines in this study with biological questions, but an mpx line was not available to us and therefore would be at least a year of work to confirm this probe is specific. Mpeg1 is a widely-used and specific marker for macrophages in the zebrafish community. We were convinced that this marker would be specific to macrophages (and not mark neutrophils), given the extensive characterization of the mpeg1:GFP transgenic line, and re-analysis of our published single cell data (Reviewer Figure 1) that shows that mpeg1 expression is limited to macrophages, and is not found in the mpx+ granulocyte population (neutrophils).

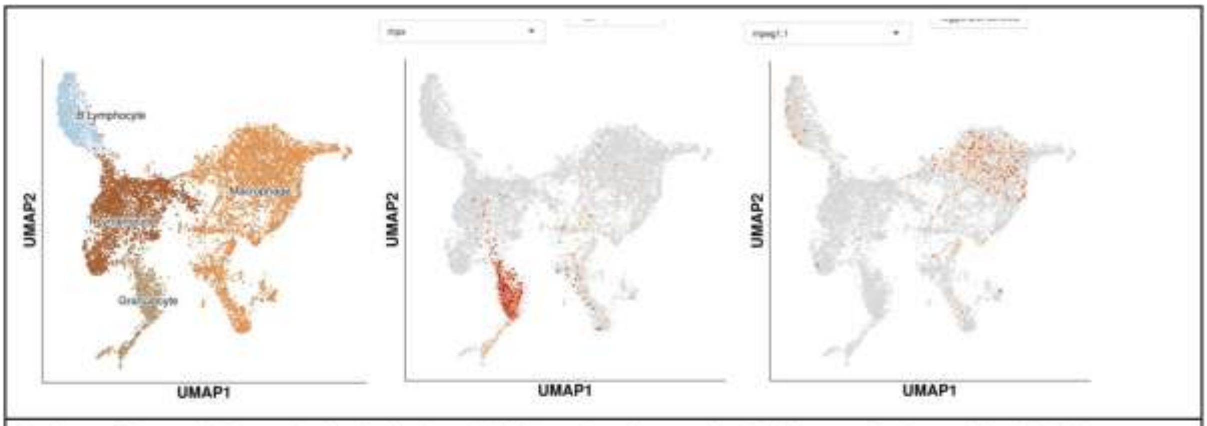

Reviewer Figure 1. Re-analysis of single-cell RNAseq data from zebrafish hearts. Cell type labels (left), mpx expression (middle), and mpeg1.1 expression (right) are shown.

- Figure 5. RNA-FISH for kdrl is not appropriate to assess neovascularization. kdrl is expressed in endocardial and vascular endothelial cells as well as in CMs (at low levels). To evaluate vascular defects, the authors should use vascular reporters and/or antibody staining for appropriate established markers (e.g., Fli, Aldh1a2, etc).

We now present data using the kdrl:GFP line, which we imaged using a GFP nanobody to boost the signal. We see only a modest defect in the ifnphi1 mutant, both in uninjured and injured zones. This is again consistent with minimal difference between wild-type and mutant at these early time points. Our study is focused on the initial response to interferon signaling. Subsequent indirect phenotypes like neovascularization are not the focus of our study and

© 2026. Published by The Company of Biologists under the terms of the Creative Commons Attribution License (https://creativecommons.org/licenses/by/4.0/).

could be the basis of a followup study. We now clarify in the Discussion that additional studies should be performed to assess neovascularization given the caveats with this experiment and the narrow scope of our investigation into vasculature.

- It would be informative to examine whether mutants display defects in CM proliferation or dedifferentiation, which could contribute to the reduced regenerative capacity observed.

Our study is focused on the initial response to interferon signaling. Subsequent indirect phenotypes like CM proliferation and dedifferentiation are not the focus of our study and could be the basis of a followup study.

We agree that this would be an interesting experiment but out-of-scope for this particular study. We now highlight this potential mechanism in the Discussion.

- Similarly, it would be interesting to assess whether collagens are upregulated in injured mutants compared with wild types.

Our study is focused on the initial response to interferon signaling. Subsequent indirect phenotypes like collagen expression are not the focus of our study and could be the basis of a followup study.

We agree that this would be an interesting experiment but out-of-scope for this particular study. We now highlight this potential mechanism in the Discussion.

Minor comments:
- Figure 1C. use "hps" (hours post sham) rather than "hpi"

We have fixed this.

- HPI and DPI are used throughout the manuscript, please standardize.

This is challenging since some of our experiments are at 2 hours post-injury, and experiments at the other extreme are 90 days post-injury. We have gone through the manuscript to make sure that we use only one term for each timepoint (i.e. referring to 3 dpi, not 72 hpi).

- The term "scar" implies permanence and should not be used for zebrafish, where fibrosis is transient. Instead, terms such as "fibrosis," "collagen accumulation," or "fibrotic tissue" are appropriate.

Thank you for this clarification - we have revised the text accordingly.

- Line 15: Please clarify the meaning of "fresh" CMs?

We have changed to "injury-responsive CMs."

- Line 96: Please clarify what "distantly related" means here.

We now explicitly describe the relationship between zebrafish and medaka as having "a common ancestor ~140 million years ago."

Reviewer 2: The manuscript by Schmid and Gagnon addresses an interesting question in cardiac regeneration, focusing on how type I interferon signalling, specifically IFNϕ1, shapes the early inflammatory response and later regenerative outcome upon zebrafish cardiac injury. The work is well executed and overall the data support a role for IFNϕ1 in driving an interferon-stimulated gene programme and in promoting early neutrophil accumulation in the injured heart. However, a few additional controls, clarifications and/or some softening of language is need to make sure the study makes a solid mechanistic contribution to how early innate signalling regulates zebrafish heart regeneration- please see points below.

© 2026. Published by The Company of Biologists under the terms of the Creative Commons Attribution License (https://creativecommons.org/licenses/by/4.0/).

We thank this reviewer for their careful reading of our manuscript and their constructive criticism. We have revised based on their comments - including new experiments and analysis - and now present an improved manuscript. A point-by-point response is below.

1. The study relies entirely on the CRISPR ifnphi1 deletion but does not directly demonstrate loss or strong reduction of ifnphi1 transcript, as shown in Figure 2E. An explanation to the observed lack of effect on ifnphi1 transcript levels should be included and the use or an orthogonal method such as HCR/RNAscope with probes designed against the mutated transcripts would be necessary to directly validate the mutant specificity.

We expected that ifnphi1 expression would be similar in cryoinjured wild-type and mutant hearts. Our zj7 mutant allele of ifnphi1 is a large in-frame deletion, which should not induce NMD-mediated degradation of the mRNA. To detect expression of wild-type and ifnphi1 mutant mRNA, we deliberately used qPCR primers that span exons 4-5, outside the zj7 deletion, to measure mRNA expression levels. Expression is quite low and variable, but this is why it should be similar across genotypes.

Several lines of evidence suggest that the zj7 allele is a null mutant. First, as previously mentioned, there is no isg15 expression after heart injury in ifnphi1 mutants (Figure 1B). Second, AlphaFold prediction of the ifnphi1 protein structure suggests that this mutation will destroy the correct fold of the protein - it deletes >30% of amino acids from the mature ifnphi1 protein sequence, eliminating or dramatically altering 3 of the 6 alpha helices that form the barrel-like central structure of this signaling protein. This can be visualized on the AlphaFold predicted structure of D. rerio ifnphi1 protein at this link https://alphafoldserver.com/fold/42d87d06e8078c20, where amino acids 30 - 78 are deleted in the mutant allele. We attempted to confirm that this mutant protein was non-functional by cloning wild-type and zj7 mutant cDNAs and injecting them into a isg15:GFP transgenic line that we have previously generated (Balla et al., *Current Biology* 2020). Unfortunately these tests were inconclusive - neither wild-type or mutant ifnphi1 was capable of activating the reporter in the early stage embryo, suggesting that the interferon signaling pathway is not present in the early embryo. This is consistent with the absence of interferon receptor expression in published zebrafish embryo datasets like Daniocell (Sur et al., *Dev. Cell* 2023). Still, these data together suggest that our zj7 ifnphi1 mutant allele is a true null allele.

Our attempts to use RNAscope to image ifnphi1 expression in injured hearts have failed - we were warned by ACDBio that the short length of these transcripts (the gene is very small) made probe design challenging, and in our hands these probes gave no signal, even in positive control samples from other tissues. For reasons we don't understand, HCR-FISH has never worked on sectioned zebrafish hearts in our hands.

2. The ifnphi1 mutant data are interpreted as a defect in neutrophil recruitment to the injury, but the description does not make it clear whether total neutrophil numbers per ventricle (injured + uninjured) differ between mutant and wild type counterparts, nor whether neutrophil density in uninjured myocardium is unchanged. Without these controls, the phenotype could equally represent reduced myelopoiesis or survival rather than altered recruitment.

We appreciate the opportunity to clarify what we mean with the term 'recruitment.' Previous publications have shown that few neutrophils are present in the uninjured homeostatic zebrafish heart. After cryoinjury, neutrophils enter the heart and accumulate at the site of injury. We now quantify the total number of neutrophils in wild-type and ifnphi1 mutant hearts and find that there are less neutrophils in injured ifnphi1 mutant hearts at both 1 and 3 days post-injury (Figure 3C and 3F). Normalized to the area of uninjured and injured zones, we find that this decreased recruitment is specific to the injured zone at both timepoints in ifnphi1 mutant hearts (Figure 3B and 3E). We deliberately chose the term recruitment to refer to this increase in the number of neutrophils in the heart overall and to the site of injury. Given that there are very few neutrophils in the heart before injury, and after injury they are found in the heart and at highest concentration within the injured zone, we feel comfortable implying a deliberate localization of neutrophils to the wound. Together with previous Figures, we suggest that this recruitment is driven at least in part by interferon signaling.

© 2026. Published by The Company of Biologists under the terms of the Creative Commons Attribution License (https://creativecommons.org/licenses/by/4.0/).

3. In the comparison of scar resolution at 30 dpi and 60 dpi it will be important to clarify whether these hearts derive from the same injury cohort.

We now clarify that these hearts derived from the same injury cohorts. To be clear, we typically injured ~20 hearts from both genotypes per day, and did injuries across multiple days to accumulate enough fish for the entire timecourse.

4. For all RNAscope figures, including supplementary panels, scale bars and injury borders must be clearly shown on the representative images. This would improve readability/clarity on the spatial distribution of signal relative to the wound and the regions used for quantification.

Drawing dashed lines to indicate the border zone interfered with the interpretability of the images, in our opinion. Instead, we added labels for the uninjured ventricle and wound in each image, and added scale bars for each image.

5. The Methods should specify the sex and approximate age range of the zebrafish used for injury experiments, whether fish were randomised to treatment and if injuries and scar scoring were performed blind to genotype.

We have clarified this information in the Methods.

Second decision letter

MS ID#: bio.062374R1

MS Title: Interferon signaling is required for early neutrophil recruitment after zebrafish heart injury

Authors: Alexis V. Schmid and James Gagnon

I have now reached a decision on the above manuscript.

The reviewer reports are shown at the bottom of this email.

As you will see, the reviewers gave favourable reports, but raised some critical points that will require amendments to your manuscript. I hope that you will be able to carry these out, because we would like to be able to accept your paper.

I don't think you need to do any additional experiments. If you tone down your conclusions and openly discuss the limitations (per reviewer 1's comments), that should be sufficient. Additionally, please do indicate the regions of injury in your micrographs. If you are concerned about such indications obscuring the images, then please generate a version of these figures that contains the outlines of the injury areas and you can include the original images (lacking injury outlines) as supplemental figures. Finally, please also explicitly mention the details of the mutant zj7 allele, such as the in-frame deletion and why you think this mitigates genetic compensation. All the good explanations and clarifications you included in the response to reviewer comments, including the reviewer figures, should be added to the revised manuscript. This will give readers a complete picture of your interesting results. If you have any questions about specific responses or modifications, don't hesitate to reach out to me for clarification.

Please upload both a 'clean' version of your Word file, along with a highlighted version clearly showing where you have made changes in the revised manuscript. Please avoid using 'Track changes' in Word files as these are lost in PDF conversion.

© 2026. Published by The Company of Biologists under the terms of the Creative Commons Attribution License (https://creativecommons.org/licenses/by/4.0/).

I should be grateful if you would also provide a point-by-point response detailing how you have dealt with the points raised by the reviewers in the 'Response to Reviewers' box. Please attend to all of the reviewers' comments. If you do not agree with any of their criticisms or suggestions please explain clearly why this is so.

Reviewer 1

Comments for the author

The authors' efforts in revising the manuscript are appreciated. However, a number of important points have not been adequately addressed.

- The mpeg transgenic line(s) and the mpeg gene are well known to be expressed in macrophages. However, the authors utilize a new mpeg probe in this manuscript, which requires validation. This is especially important since it is widely recognized that discriminating background noise is a significant challenge with these types of stainings. Figure 3 shows mutants in an mpx:GFP background; co-staining in this background would allow the authors to discriminate non-specific signals originating from neutrophils and further support probe specificity. At a bare minimum, the aforementioned limitations should be explicitly acknowledged.

- Injury size, section orientation, and injured area: The injured areas shown throughout the manuscript are extremely variable and fall below the established standards in the field. For instance, in Figure 3A, the +/+ injury is significantly larger than the one shown in the mutant heart, which appears superficial at best. Similar discrepancies are observable in Figures 3D and 5F. This inconsistency affects quantifications and could even explain some of the observed variability. Furthermore, injured areas must be delineated in every image. Delineating the injuries is a minimum requirement so the reader can at least visualize these differences.

- Neutrophil recruitment claims: The authors cannot exclude the possibility that the neutrophil phenotype may be due to other defects, such as neutrophil accumulation or defective reverse dynamics. While it is understandable that neutrophil dynamic visualization cannot be performed, the authors should refrain from using the term "recruitment" in absence of such assays. Similarly, the primary claim in the title and throughout the manuscript (that Interferon signaling promotes early neutrophil recruitment) is not supported by the data. No gain-of-function data are provided. Thus, there is no experimental evidence showing that manipulating interferon signaling increases or promotes neutrophil recruitment. The loss-of-function data provided suggests the signaling is necessary or required. If no experimental evidence can be provided to support the this claim, the authors should modify the text throughout the manuscript to better reflect the obtained results and their interpretation.

- Revascularization phenotypes: The kdrl line and probe are not coronary-specific, and the regions magnified in the figures correspond to the endocardium. Therefore, coronary-specific phenotypes cannot be assessed with the data provided. Unless other specific markers are available, the authors should refer to the "cardiac endothelium" rather than the coronary vasculature.

Reviewer 2

Comments for the author

The revised manuscript is substantially improved and most of my concerns have been addressed. The added analyses and clarifications strengthen the study and support the conclusion that IFNϕ1-dependent interferon signalling promotes early neutrophil recruitment after zebrafish heart injury.

One important issue still needs to be made clearer in the manuscript. The interpretation of the zj7 allele remains under-explained in the main text and it is an important point to clarify to the non-specialist reader. The manuscript should state explicitly that the deletion is in-frame, that continued transcript detection is therefore not unexpected and that the allele is inferred to be LOF on the basis of loss of injury-induced isg15 but is not directly demonstrated to be null. This point is important for interpretation of the mutant phenotype and should be fully included in the manuscript rather than left mainly in the rebuttal.

© 2026. Published by The Company of Biologists under the terms of the Creative Commons Attribution License (https://creativecommons.org/licenses/by/4.0/).

The revised RNAscope figures are improved but the wound region is still not consistently obvious to the reader. Because the analysis depends on distinguishing injured from uninjured areas, the representative images should show this more clearly, specially thinking of non-specialist readers. Dashed outlines would be the most straightforward solution. But if the authors prefer not to add them to the main figure, a supplementary overlay showing dashed lines in the border used for quantification should be included.

Reviewer's Responses to Questions

Experimental quality
Are experiments performed using appropriate methods that will answer the question (or test the hypothesis or support the observations) posed by the authors? Is the right tool used for the job?
If 'No', please indicate reasons in Comments for Author box below.
Reviewer #1:
- No

Reviewer #2:
- Yes

Were the data analyzed using appropriate statistical tests?
If 'No', please indicate reasons in Comments for Author box below.
Reviewer #1:
- Yes

Reviewer #2:
- Yes

Reproducibility
Were experiments performed using adequate number of biological replicates?
If 'No', please indicate reasons in Comments for Author box below.
Reviewer #1:
- Yes

Reviewer #2:
- Yes

Is there sufficient raw data to assess the rigor of the analysis?
If 'No', please indicate reasons in Comments for Author box below.
Reviewer #1:
- Yes

Reviewer #2:
- Yes

Does the methods section provide sufficient detail to permit reproducibility?
If 'No', please indicate reasons in Comments for Author box below.
Reviewer #1:
- Yes

Reviewer #2:
- Yes

© 2026. Published by The Company of Biologists under the terms of the Creative Commons Attribution License (https://creativecommons.org/licenses/by/4.0/).

Completeness
Are the manuscript's conclusions supported by the data?
If 'No', please indicate reasons in Comments for Author box below.
Reviewer #1:
- No

Reviewer #2:
- Yes

Scholarship
Do the authors cite and discuss the merits of data that would argue for and against their conclusion?
If 'No', please indicate reasons in Comments for Author box below.
Reviewer #1:
- Yes

Reviewer #2:
- Yes

Does the manuscript title & abstract accurately reflect the contents of the manuscript, without hyperbole?
If 'No', please indicate reasons in Comments for Author box below.
Reviewer #1:
- No

Reviewer #2:
- Yes

Second revision

Author response to reviewers' comments

Reviewer 1: The authors' efforts in revising the manuscript are appreciated. However, a number of important points have not been adequately addressed.

- The mpeg transgenic line(s) and the mpeg gene are well known to be expressed in macrophages. However, the authors utilize a new mpeg probe in this manuscript, which requires validation. This is especially important since it is widely recognized that discriminating background noise is a significant challenge with these types of stainings. Figure 3 shows mutants in an mpx:GFP background; co-staining in this background would allow the authors to discriminate non-specific signals originating from neutrophils and further support probe specificity. At a bare minimum, the aforementioned limitations should be explicitly acknowledged.

Thank you for clarifying your concerns with this reagent. We may have misunderstood your comment from the previous round of review. It is possible, although we think unlikely, that non-specific mpeg1.1 probe staining of neutrophils contributes to the fluorescence we observe and assign to macrophages in Figure 4. We used this RNAscope probe in a previous publication (Carey et al., *Biology Open* 2024). We believe that this RNAscope probe is specific to macrophages, given what we know about cell-type specific mpeg1.1 expression and previous RNA hybridization studies, etc. However, we cannot complete the requested experiment for verification of probe specificity (RNAscope + immunostaining for GFP in a homozygous mutant transgenic background) given a combination of technical reagent and animal limitations.

Therefore, we have clarified this point in the Methods:

© 2026. Published by The Company of Biologists under the terms of the Creative Commons Attribution License (https://creativecommons.org/licenses/by/4.0/).

“While RNAscope is a commonly-used method for RNA-FISH, it should be acknowledged that the specificity of the probes used in these experiments have not been explicitly validated.”

- Injury size, section orientation, and injured area: The injured areas shown throughout the manuscript are extremely variable and fall below the established standards in the field. For instance, in Figure 3A, the +/+ injury is significantly larger than the one shown in the mutant heart, which appears superficial at best. Similar discrepancies are observable in Figures 3D and 5F. This inconsistency affects quantifications and could even explain some of the observed variability. Furthermore, injured areas must be delineated in every image. Delineating the injuries is a minimum requirement so the reader can at least visualize these differences.

Thank you for this suggestion. We have replaced the image of the injured mutant heart in Figure 3A with a sample that more closely matched the size of the compared wildtype heart injury. We now delineate the injury border locations in all fluorescently-stained heart sections to make the injury location more obvious. Heart cryoinjury following well-established methods is inevitably variable between individuals in our study and in many previously-published reports. We use area normalization and replicates to account for this variability.

- Neutrophil recruitment claims: The authors cannot exclude the possibility that the neutrophil phenotype may be due to other defects, such as neutrophil accumulation or defective reverse dynamics. While it is understandable that neutrophil dynamic visualization cannot be performed, the authors should refrain from using the term "recruitment" in absence of such assays. Similarly, the primary claim in the title and throughout the manuscript (that Interferon signaling promotes early neutrophil recruitment) is not supported by the data. No gain-of-function data are provided. Thus, there is no experimental evidence showing that manipulating interferon signaling increases or promotes neutrophil recruitment. The loss-of-function data provided suggests the signaling is necessary or required. If no experimental evidence can be provided to support the this claim, the authors should modify the text throughout the manuscript to better reflect the obtained results and their interpretation.

We now change the title to “Interferon signaling is required for early neutrophil recruitment after zebrafish heart injury.”

As this reviewer points out, since we did LOF experiments, not GOF experiments, we can support claims about necessity but not sufficiency. In normal conditions, neutrophils rapidly move to the heart injury site (previous literature). When interferon signaling is lost, neutrophils are not found in the heart injury site (our data). We have now revised the title to be more in line with our LOF data.

We believe that “neutrophil recruitment” is a commonly used phrase in the literature, which to our understanding is an umbrella term that may be directed by several possible dynamic cellular processes (chemokine-directed migration, retention of circulating cells, delayed reverse dynamics, others). Picking two examples from many in the literature, in one seminal zebrafish heart regeneration study on neutrophils, they refer to similar snapshot imaging data as “neutrophil recruitment” (Figure 3, Lai et al., *eLife* 2017). More recently, another excellent study on zebrafish neutrophil behavior after heart injury also refers to similar snapshot imaging data as neutrophil recruitment (Figure 1, Goumenaki et al., *Nature Cardiovascular Research* 2024). The second paper went further by characterizing a chemokine involved in neutrophil recruitment, which further narrows the mechanism of recruitment at least partially to chemotaxis, although it does not rule out contributions from other processes.

Whether neutrophils are migrating to the wound through chemotaxis, accumulating there by increased retention, or staying in the wound longer (also possible, but less likely at 1 dpi given previous studies), we still believe the word ‘recruitment’ succinctly captures a spectrum of mechanisms that may underlie this phenomenon.

We now clarify these points in the Discussion, notably that 1) ‘recruitment’ could be generated by several mechanisms, 2) our data does not differentiate between these possibilities, and 3) there are some key experiments that could address the underlying mechanisms.

© 2026. Published by The Company of Biologists under the terms of the Creative Commons Attribution License (https://creativecommons.org/licenses/by/4.0/).

- Revascularization phenotypes: The kdrl line and probe are not coronary-specific, and the regions magnified in the figures correspond to the endocardium. Therefore, coronary-specific phenotypes cannot be assessed with the data provided. Unless other specific markers are available, the authors should refer to the "cardiac endothelium" rather than the coronary vasculature.

Thank you for catching this inadvertent omission in our revision. We have revised throughout.

Reviewer 2: The revised manuscript is substantially improved and most of my concerns have been addressed. The added analyses and clarifications strengthen the study and support the conclusion that IFNϕ1-dependent interferon signalling promotes early neutrophil recruitment after zebrafish heart injury.

One important issue still needs to be made clearer in the manuscript. The interpretation of the zj7 allele remains under-explained in the main text and it is an important point to clarify to the non-specialist reader. The manuscript should state explicitly that the deletion is in-frame, that continued transcript detection is therefore not unexpected and that the allele is inferred to be LOF on the basis of loss of injury-induced isg15 but is not directly demonstrated to be null. This point is important for interpretation of the mutant phenotype and should be fully included in the manuscript rather than left mainly in the rebuttal.

Thank you for catching this inadvertent omission in our revision. We now discuss this allele in more detail in the Results and Methods.

The revised RNAscope figures are improved but the wound region is still not consistently obvious to the reader. Because the analysis depends on distinguishing injured from uninjured areas, the representative images should show this more clearly, specially thinking of non-specialist readers. Dashed outlines would be the most straightforward solution. But if the authors prefer not to add them to the main figure, a supplementary overlay showing dashed lines in the border used for quantification should be included.

We now delineate the injury border locations in all fluorescently-stained heart sections to make the injury location more obvious.

<u>Third decision letter</u>

MS ID#: bio.062374R2

MS Title: Interferon signaling is required for early neutrophil recruitment after zebrafish heart injury

Authors: Alexis V. Schmid and James Gagnon

Thank you for submitting a second revised version of your manuscript. I've read through it and you have addressed all the comments adequately. I am happy to tell you that your manuscript has been accepted for publication in Biology Open, pending our standard publication integrity checks. It was accepted on 8th May 2026.

© 2026. Published by The Company of Biologists under the terms of the Creative Commons Attribution License (https://creativecommons.org/licenses/by/4.0/).
